# Dynamic transcriptomic responses reveal candidate defense genes against *Spongospora subterranea* f. sp. s*ubterranea* and Potato mop-top virus infection

**DOI:** 10.3389/fpls.2026.1799568

**Published:** 2026-06-11

**Authors:** Samodya K. Jayasinghe, Natalia Moroz, Stephen P. Ficklin, Kiwamu Tanaka

**Affiliations:** 1 Department of Plant Pathology, Washington State University, Pullman, WA, United States; 2 Department of Horticulture, Washington State University, Pullman, WA, United States

**Keywords:** plant defense, potato, Potato mop-top virus, powdery scab, transcriptomics

## Abstract

Powdery scab, caused by *Spongospora subterranea* f.sp. *subterranea* (*Sss*), is persistent soilborne disease of potato. *Sss* is also a vector for Potato mop-top virus (PMTV), posing another significant threat to tuber quality and marketability. Although currently available cultivars exhibit partial resistance to both *Sss* and PMTV, the genetic mechanisms of resistance remain poorly understood. In this study, we investigated the molecular basis of resistance to *Sss* and PMTV using a transcriptomics approach. Early infection time-course RNA-seq data were analyzed from three potato cultivars exhibiting differential susceptibility. Integrative analysis of differential gene expression (DGE) and gene co-expression networks (GCN) identified 80 high-confidence candidate genes associated with defense responses, comprising 52 genes associated with *Sss* infection and 28 with PMTV infection. These included putative immune receptor genes such as nucleotide-binding leucine-rich repeat (NLR) proteins and leucine-rich repeat receptor-like kinases (LRR-RLKs), which function as resistance (R) genes and pattern recognition receptors (PRRs), respectively. Additional candidates included phytohormone-related genes and those encoding lipoxygenase (LOX), glutathione-S-transferase (GST), and Bet v I family protein (PR10). These findings advance our understanding of host defense mechanisms against *Sss* and PMTV and provide molecular targets for resistance breeding and genetic improvement of commercially important potato cultivars.

## Introduction

Potato powdery scab is a globally important soilborne disease caused by the plasmodiophorid protist pathogen *Spongospora subterranea* f. sp. *subterranea* (*Sss*). The pathogen primarily infects underground plant structures, producing wart-like pustules on tubers and gall-like outgrowths on roots and stolons, while aboveground symptoms are rarely observed (Mora-Romero et al., 2022). Although field symptoms may be inconspicuous, the cumulative effects can be substantial, as severe root galling impairs water and nutrient uptake, leading to reduced plant vigor and yield (Merz and Falloon, 2009; Kamal et al., 2024). Yield losses caused by *Sss* can be substantial, ranging from significant economic losses of approximately $13.4 million annually in Australia (Wilson, 2016) to yield reductions of 5–12 t ha^−1^ in the U.S. (Nitzan et al., 2008), and up to a 42% decrease in tuber weight per plant (Falloon et al., 2016). Tuber blemishes further reduce market value through postharvest culling, particularly in the fresh market.

Beyond its direct pathogenic effects, *Sss* is the only known vector of Potato mop-top virus (PMTV), which causes tuber necrosis and renders tubers unsuitable for consumption. PMTV is classified as a quarantine pathogen in several countries (Lee et al., 2017; EPPO Global Database), presenting further challenges for international shipment of seed and table potatoes and intensifying the economic significance of *Sss* (Duellman et al., 2022).

Management of *Sss* remains challenging. Effective chemical control strategies are currently unavailable, and cultural practices such as crop rotation are often ineffective due to the broad host range of *Sss* (Qu and Christ, 2006). Moreover, long-lived resting spores (sporosori) enable persistence in infested fields for more than decade, making eradication impractical. Consequently, sustainable production of clean seed tubers and a healthy crop depend on host resistance. However, despite the clear need, genomic knowledge underpinning resistance to *Sss* and PMTV remains limited.

Commercial cultivars show a wide spectrum of susceptibility to *Sss* (Nitzan et al., 2009; Jayasinghe et al., 2026), ranging from highly susceptible to moderately tolerant, although highly resistant cultivars have not yet been identified. These phenotypic differences strongly suggest a genetic basis for resistance (Falloon et al., 2003; Yu et al., 2023). Indeed, recent studies have reported differential gene regulation among cultivars with contrasting levels of susceptibility, indicating the resistance to *Sss* is likely quantitative and polygenic (Jayasinghe et al., 2026; Lekota et al., 2019). Therefore, we hypothesize that multiple genetic factors and regulatory mechanisms govern resistance and susceptibility to *Sss* and PMTV in potato.

To investigate these mechanisms, we performed transcriptomic analyses on three potato cultivars with contrasting susceptibility levels, following early infection with either non-viruliferous *Sss* or PMTV-carrying *Sss*. This experimental framework enables the dissection of host responses to *Sss* alone and to *Sss*-mediated PMTV infection. While previous transcriptomic studies have focused on tubers, no studies have examined root tissues (Lekota et al., 2019). In the present study, we employed a hairy root culture system to investigate early infection stages using transcriptomics. Furthermore, to gain deeper insight into resistance-associated molecular processes, we integrated differential gene expression (DGE) analysis with gene co-expression network (GCN) analysis. While DGE identifies genes whose expression changes significantly upon infection, GCN analysis reveals coordinated transcriptional modules, hub genes, and potential regulatory networks. The DGE-GCN combined approach, therefore, enhances biological interpretation, capturing both responsive genes and their functional context. Through this integrative framework, we identified robust candidate genes potentially contributing to resistance or susceptibility during the early stages of *Sss* and PMTV infection, providing valuable genomic resources for resistance breeding and advancing understanding of host-pathogen interactions underlying these economically significant potato diseases.

## Materials and methods

### Inoculum preparation

Potato tubers of the cultivar Shepody exhibiting characteristic powdery scab symptoms were collected from commercial potato fields in central Washington and eastern Oregon areas. Potato skin peels containing lesioned tissues were air-dried and finely ground to obtain sporosori-containing powder, which served as viruliferous and nonviruliferous *Sss* inoculum stocks. To eliminate potential microbial contaminants and plant debris, the inoculum preparation from the powdery scab powder mix was purified using the methods described previously (Moroz et al., 2024; Jayasinghe et al., 2025). The purified sporosori were suspended in Hoagland’s solution, and the final concentration was adjusted to 1-5 × 10^5^ sporosori per milliliter prior to use.

### Potato hairy root cultures

Hairy root cultures were generated from three potato cultivars, Shepody (SH), Russet Burbank (RB), and Premier Russet (PR), using methods described in Jayasinghe et al. (2025). Root tip segments (1 cm) were excised at the tip of the hairy root and transferred individually into wells of 6-well plates containing MS agar medium. Cultures were maintained in the dark at 16°C for 14 days to allow establishment and growth. Subsequently, each well was inoculated with 100 µL of sporosori suspension, applied directly onto the roots. Inoculated roots were incubated in the dark at 16°C until the respective sampling times. Mock controls were treated similarly, using Hoagland’s solution without sporosori.

### Sample collection and RNA extraction

Root tissues were collected from inoculated and mock-treated roots of all three cultivars at 3, 6, 12, 24, 48, and 72 h post-inoculation. These time points were chosen to capture host-pathogen interactions during the early stages of infection, based on our preliminary data (**Supplementary Figure 1**) and previous studies (Jayasinghe et al., 2025; Yu et al., 2023; Moroz et al., 2017) indicating that early infection and host defense response occur within 72 h after *Sss* inoculation. Immediately following removal from MS medium, each root segment was surface-sterilized by dipping it in 75% ethanol and rinsed three times with sterile water. Samples were briefly blotted on sterile filter paper, transferred to a sterile microcentrifuge tube, and snap-frozen in liquid nitrogen. The entire handling process was completed within 8–15 sec for each sample. Sampling was performed in two separate experiments, with inoculation and harvests conducted at the same time of day in both experiments to control circadian influences on gene expression. Four biological replicates were collected for each cultivar × treatment × time point combination. All root samples were stored in -80 °C until RNA extraction.

Total RNA was extracted using the Quick-RNA MiniPrep kit (Zymo Research, Cat no. R1055) according to the manufacturer’s instructions. Genomic DNA contamination was removed by on-column DNase I treatment. Sequencing libraries were prepared and sequenced on the Illumina NovaSeq platform (2 × 150 bp paired-end) by Novogene Corporation Inc. (CA, USA).

### Post-alignment process, outlier filtering, and noise correction

Raw reads were aligned to the *Solanum tuberosum* Group Phureja DM1–3 reference genome (v6.1). Reference genome and annotation data were obtained from the Phytozome (Goodstein et al., 2012). Transcript quantification and generation of the gene expression matrix were conducted using the GEMmaker nextflow workflow (Hadish et al., 2022).

Initial exploratory analyses revealed the presence of batch effects and technical noise. Therefore, a structured data cleaning workflow was performed. First, the gene Soltu.DM.09G002330.1 (heat shock 70 kDa protein cognate 1-related), which exhibited disproportionally high expression relative to all other genes and artificially skewed sample correlations, was removed from the matrix. Next, pairwise scatter plots were generated for each cultivar and time point (**Supplementary Figure 2a**), and correlation coefficients were calculated. For each pairwise comparison, the angle of the regression line relative to the x-axis was measured following equalization of axis scaling. These angles were plotted against the corresponding correlation coefficients, and biological replicates exhibiting a correlation coefficient more than 0.85 and a regression angle of 40° or higher against their replicate set were retained for downstream analysis (**Supplementary Figure 2b**).

Following outlier removal, residual technical noise and batch effects were corrected using the R package noisyR (v1.0.0) with a Spearman correlation coefficient-based model (Moutsopoulos et al., 2021). Noise removal with noisyR was performed to account for variability among biological replicates. The resulting cleaned expression matrix was subsequently used for differential gene expression (DGE) and gene co-expression network (GCN) analyses. All data generated in this study have been deposited in the Mendeley data repository (Jayasinghe and Tanaka, 2026; DOI: 10.17632/tv6mjd9c95.1).

### Differential gene expression analysis

Differential gene expression analysis (DGE) was conducted separately for each cultivar using quality-checked reads and the edgeR package in R (Robinson et al., 2010). For each cultivar, comparisons between untreated controls and *Sss*-inoculated samples were used to identify potato genes responsive to *Sss* infection. To investigate host responses to viral infection, gene expression profiles from viruliferous *Sss-*inoculated samples were compared with those from non-viruliferous *Sss-*inoculated samples. All comparisons were conducted independently at each sampling time point. Differentially expressed genes (DEGs) were identified using edgeR (v4.6.1), using generalized linear model. The model was fitted using glmFit, and differential expressions were tested using glmLRT. Genes with Benjamini-Hochberg adjusted p- value threshold of p < 0.05, and log_2_ fold change (logFC) threshold of ±1, were considered as differentially expressed. Functional enrichment of DEGs was conducted using FUNC-E (Ficklin et al., 2023), with a p-value cutoff of 0.05.

### Gene co-expression network analysis

Gene co-expression network (GCN) analysis was performed using the expression data processed with the noisyR pipeline. Two independent networks were generated: (i) mock-inoculated and non-viruliferous *Sss*-inoculated samples; and (ii) viruliferous and non-viruliferous *Sss*-inoculated samples. Networks were constructed using the Weighted Gene Co-expression Network Analysis (WGCNA) package in R (v1.73) (Langfelder and Horvath, 2008). The networks were generated using Pearson correlation and with a minimum cluster size of 50.

Module-trait association analysis was then performed to identify modules significantly correlated with the traits of interest, i.e. cultivar-treatment combinations. Modules with Pearson correlation coefficients ≥ +0.6 or ≤ -0.6 and P < 0.05 with a given trait were considered significant, and the most strongly associated module for each cultivar was selected for downstream analysis.

### Candidate gene identification

Candidate resistance- or susceptibility-associated genes were identified by comparing DEGs identified via EdgeR with GCN modules for each cultivar-treatment combination. Only upregulated DEGs were considered when the module-trait correlation was positive, whereas only downregulated DEGs were considered when the correlation was negative.

To prioritize the most biologically relevant candidates, the top 20% highest-ranked genes from each module identified from module trait correlation analysis were selected based on ranking using intramodular connectivity and module membership scores (Zhang et al., 2022). We considered the top 20% ranked genes as “hub” genes. The shared genes between these hub genes and the corresponding DEGs were defined as priority candidates.

### Reverse transcription-quantitative polymerase chain reaction (RT-qPCR)

RT-qPCR was performed to validate the expression of eight selected genes using previously described methods and conditions (Jayasinghe et al., 2025). cDNA was synthesized using 1 µg of total RNA in a 20-µL reverse transcription reaction using iScript kit (Bio-Rad) according to the manufacturer’s instructions. The primer sequences used in this study are listed in **Supplementary Table 1**.

## Results

### Batch effect and noise correction

To elucidate regulatory mechanisms underlying resistance and susceptibility to *Sss* and PMTV in potato, we performed transcriptomics and analyzed a comprehensive dataset derived from three cultivars with contrasting resistance phenotypes: Shepody (SH) as highly susceptible, Russet Burbank (RB) as moderately susceptible/resistant, and Premier Russet (PR) as relatively resistant. These cultivars have been well characterized for their responses to both *Sss* and PMTV in previous studies (Bittara et al., 2016; Falloon et al., 2003; Nitzan et al., 2008; Novy et al., 2008; Domfeh et al., 2015; Yellareddygari et al., 2018) and are consistent with our pilot data using a pot assay (**Supplementary Figure 3**), which show differences in root gall numbers among the three cultivars, supporting their contrasting susceptibilities under study conditions. Hairy root tissue was inoculated with mock solution, non-viruliferous *Sss*, or PMTV-carrying *Sss*, enabling assessment of host responses to *Sss* alone versus *Sss-*mediated PMTV transmission. Total RNA was extracted from 216 biological samples collected across six-time points, followed by Illumina-based RNA sequencing. After initial quality control of the sequencing data, 182 samples remained; subsequent removal of poorly correlated biological replicates yielded a final dataset of 145 samples. The 3 h time point was discarded due to high variability among replicates, which reduced the number of samples to 121. Following noisyR-based noise removal, data quality improvement and sample clustering were confirmed using PCA (**Supplementary Figure 4**-**S9**). The curated dataset was subsequently used for DEG and GCN analyses. The entire workflow of this transcriptomic study is shown in **Supplementary Figure 10**. All significantly differentially expressed genes (DEGs) are provided in Supplementary Data 1 (associated with Sss infection) and Supplementary Data 2 (associated with PMTV infection), including FC, CPM, and FDR values. To validate the RNA-seq data, eight genes associated with *Sss* infection were selected and analyzed by RT-qPCR. Log_2_FC values were calculated for each gene based on RT-qPCR data and plotted against the corresponding RNA-seq Log_2_FC values (**Supplementary Figure 11**). The results showed a reasonable correlation between RNA-seq and RT-qPCR data, supporting the reliability of the candidate genes identified through the RNA-seq analysis.

### Differential gene expression associated with *Sss* infection

**Figure 1:**
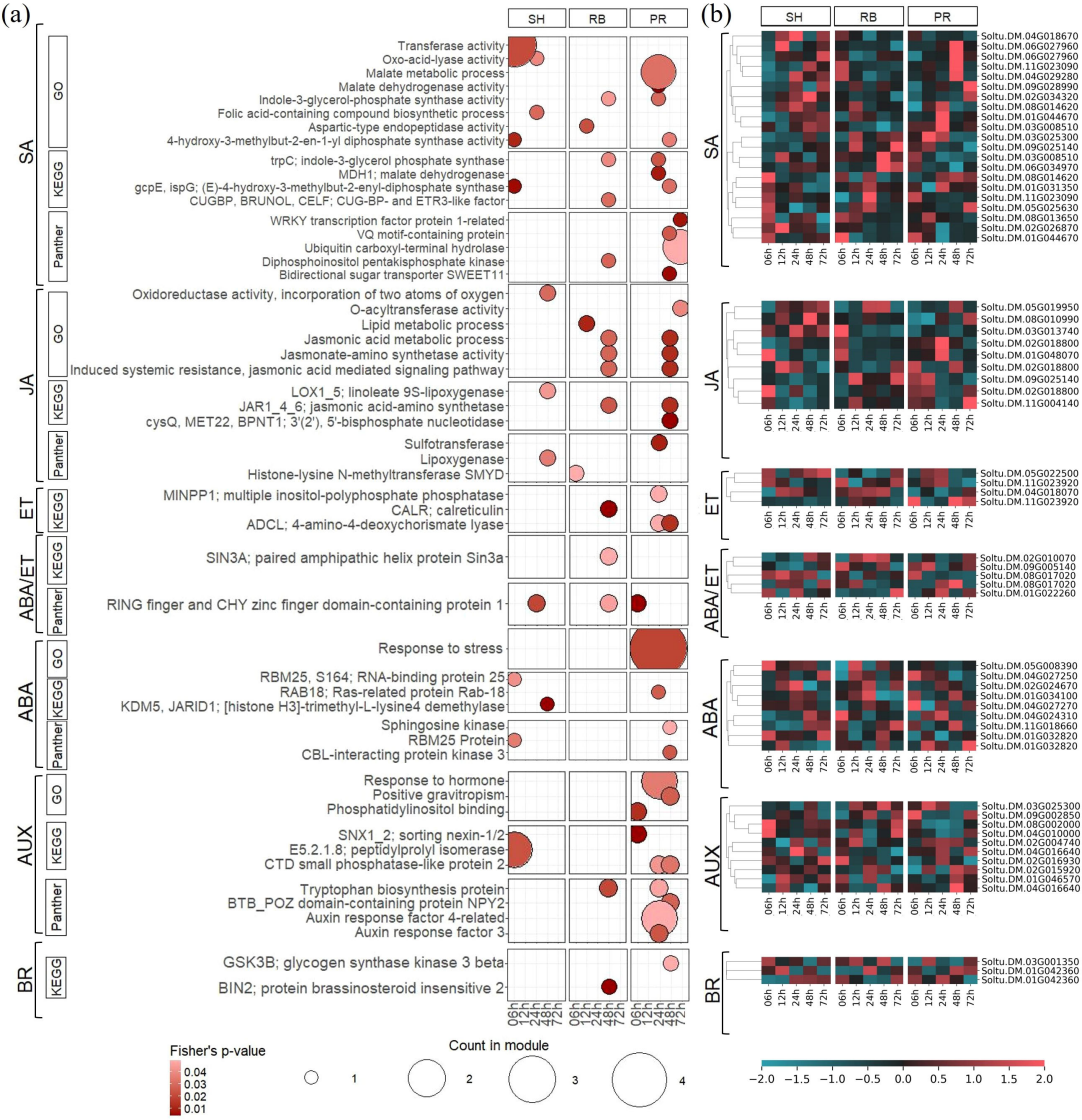
Functional enrichments of upregulated differentially expressed genes (DEGs) associated with *Sss* infection in phytohormone-related pathways. **(a)** Bubble plots showing significantly enriched, upregulated functional terms from Gene Ontology (GO), Kyoto Encyclopedia of Genes and Genomes (KEGG), and PANTHER ontology (Panther) analyses. **(b)** Heatmaps showing expression patterns of genes with each enriched functional category, identified as responsive to *Sss* infection through differential gene expression (DGE) analysis. The x-axis indicates the time points of sample collection post-pathogen inoculation across different potato cultivars. SA, salicylic acid; JA, jasmonic acid; ET, ethylene; ABA, abscisic acid; AUX, auxin; BR, brassinosteroids; SH, Shepody; RB, Russet Burbank; PR, Premier Russet.

**Figure 2:**
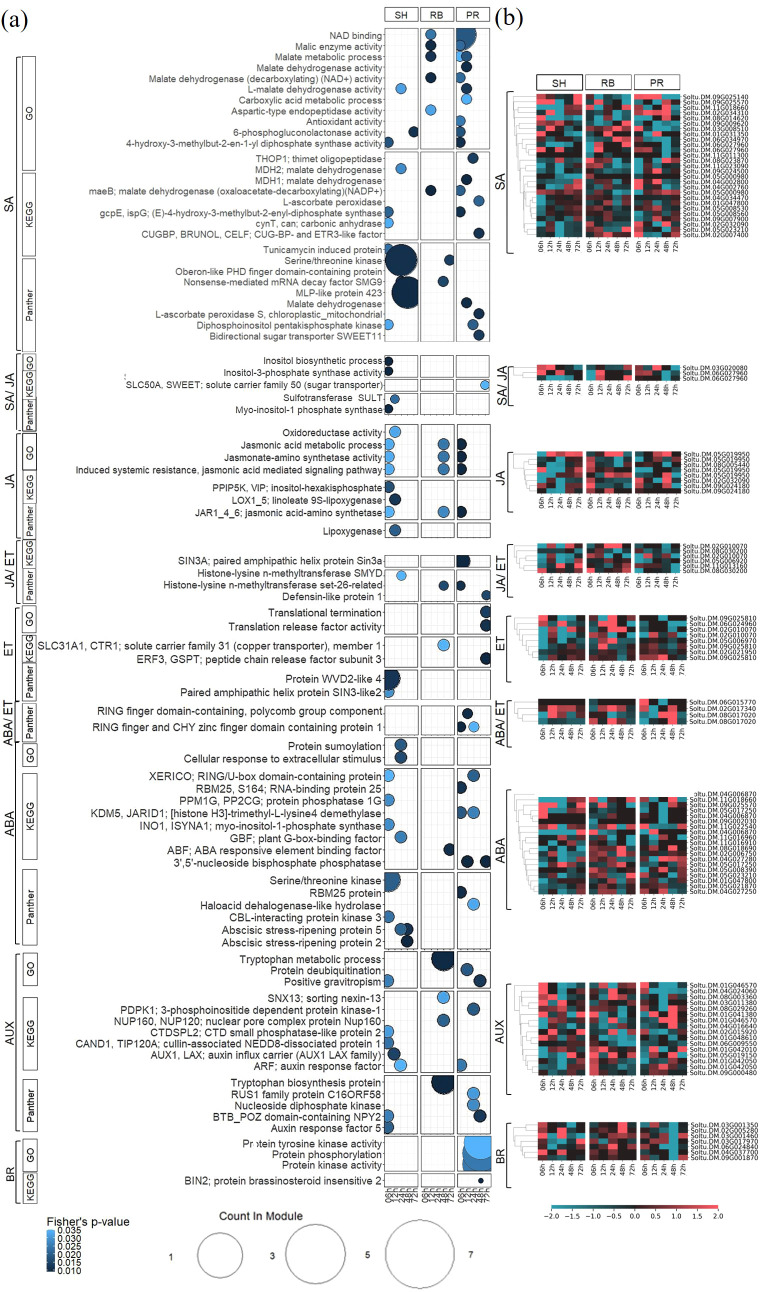
Functional enrichments of downregulated differentially expressed genes (DEGs) associated with *Sss* infection in phytohormone-related pathways. **(a)** Bubble plots showing significantly enriched, upregulated functional terms from Gene Ontology (GO), Kyoto Encyclopedia of Genes and Genomes (KEGG), and PANTHER ontology (Panther) analyses. **(b)** Heatmaps showing expression patterns of genes with each enriched functional category, identified as responsive to *Sss* infection through differential gene expression (DGE) analysis. The x-axis indicates the time points of sample collection post-pathogen inoculation across different potato cultivars. SA, salicylic acid; JA, jasmonic acid; ET, ethylene; ABA, abscisic acid; AUX, auxin; BR, brassinosteroids; SH, Shepody; RB, Russet Burbank; PR, Premier Russet.

Comparisons between mock-treated samples and non-viruliferous *Sss*-inoculated samples identified 1,783 DEGs responsive to *Sss* infection (1,043 in SH, 361 in RB, and 765 in PR). Of these, 1,248 were upregulated (668 in SH, 154 in RB, and 575 in PR), while 873 were downregulated (473 in SH, 226 in RB, and 297 in PR). Functional enrichment analysis using GO, KEGG, and Panther ontologies revealed pronounced activation of salicylic acid (SA)-associated pathway in PR (**Figure 1**), whereas SA-related terms were significantly downregulated in SH (**Figure 2**), suggesting enhanced SA-mediated defense signaling in the resistant cultivar and suppression in the susceptible cultivar. Auxin-associated genes were also upregulated in PR, whereas other phytohormone pathways (jasmonic acid, ethylene, abscisic acid, brassinosteroids, cytokinin, and gibberellins) did not show pronounced cultivar-specific enrichment patterns (**Figure 1** and **2**; **Supplementary Figures 12**, **S13**).

Defense-associated signaling components were differentially regulated across cultivars. RPM1-interacting protein 4 (RIN4), a key defense regulator, was upregulated in PR, but downregulated in RB, with no significant change in SH (**Supplementary Figures 14**, **S15**). Several genes encoding leucine-rich repeat (LRR) domain-containing proteins were differentially expressed in SH, with both up- and downregulated members detected. In addition, wall-associated receptor kinase-like 21 (WAKL21) and serine/threonine protein kinase (SMG1) were significantly upregulated in PR, indicating potential reinforcement of receptor-mediated defense signaling (**Supplementary Figure 14**).

Cell wall reinforcement is another hallmark of resistant responses. Lignin biosynthesis-related categories, including lignin catabolism and laccase, were significantly upregulated in PR (**Supplementary Figure 16**), whereas SH showed downregulation of lignin- and cell wall-related genes, including those associated with cell wall biogenesis and expansin B1-like protein (**Supplementary Figure 17**). These contrasting trends suggest structural reinforcement in PR but cell wall weakening in SH following *Sss* infection.

Reactive oxygen species (ROS), cell death, and calcium signaling contributed to defense responses across cultivars. ROS-associated terms (**Supplementary Figures 18**, **S19**) were sporadically upregulated at 6 h and in SH and at 72 h in RB. Cell death-associated terms (**Supplementary Figures 20**, **S21**) were predominantly downregulated in both SH and PR, suggesting suppression of extensive tissue collapse. Calcium signaling components displayed strong cultivar specificity. PR exhibited consistent upregulation of calcium-related processes, whereas SH predominantly showed downregulation (**Supplementary Figures 22**, **S23**), further highlighting robust signal transduction in resistant backgrounds.

### Differential gene expression associated with PMTV infection

**Figure 3:**
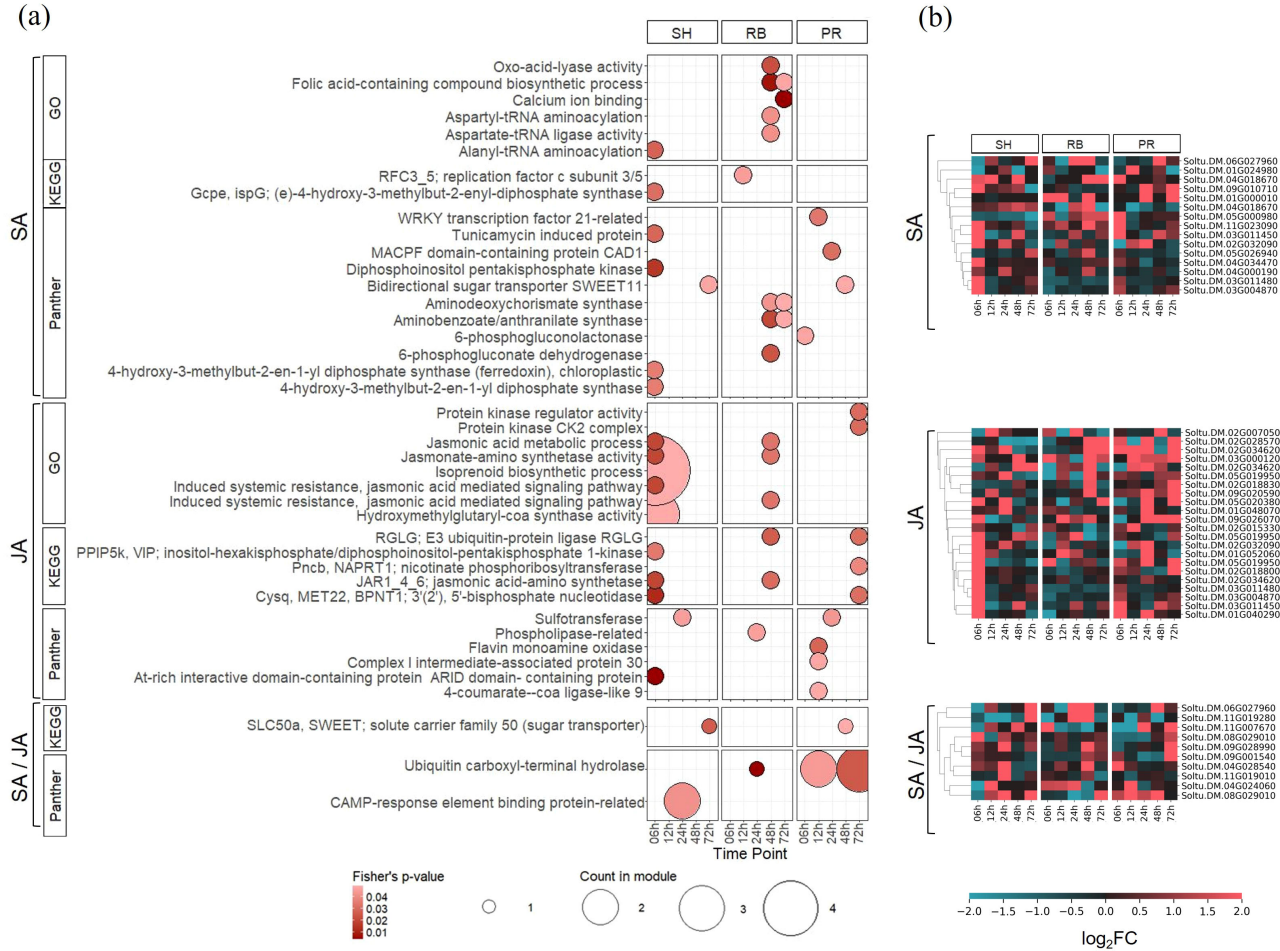
Functional enrichments of upregulated differentially expressed genes (DEGs) associated with PMTV infection in phytohormone-related pathways. **(a)** Bubble plots showing significantly enriched, upregulated functional terms from Gene Ontology (GO), Kyoto Encyclopedia of Genes and Genomes (KEGG), and PANTHER ontology (Panther) analyses. **(b)** Heatmaps showing expression patterns of genes with each enriched functional category, identified as responsive to PMTV infection through differential gene expression (DGE) analysis. The x-axis indicates the time points of sample collection post-pathogen inoculation across different potato cultivars. SA, salicylic acid; JA, jasmonic acid; SH, Shepody; RB, Russet Burbank; PR, Premier Russet.

**Figure 4:**
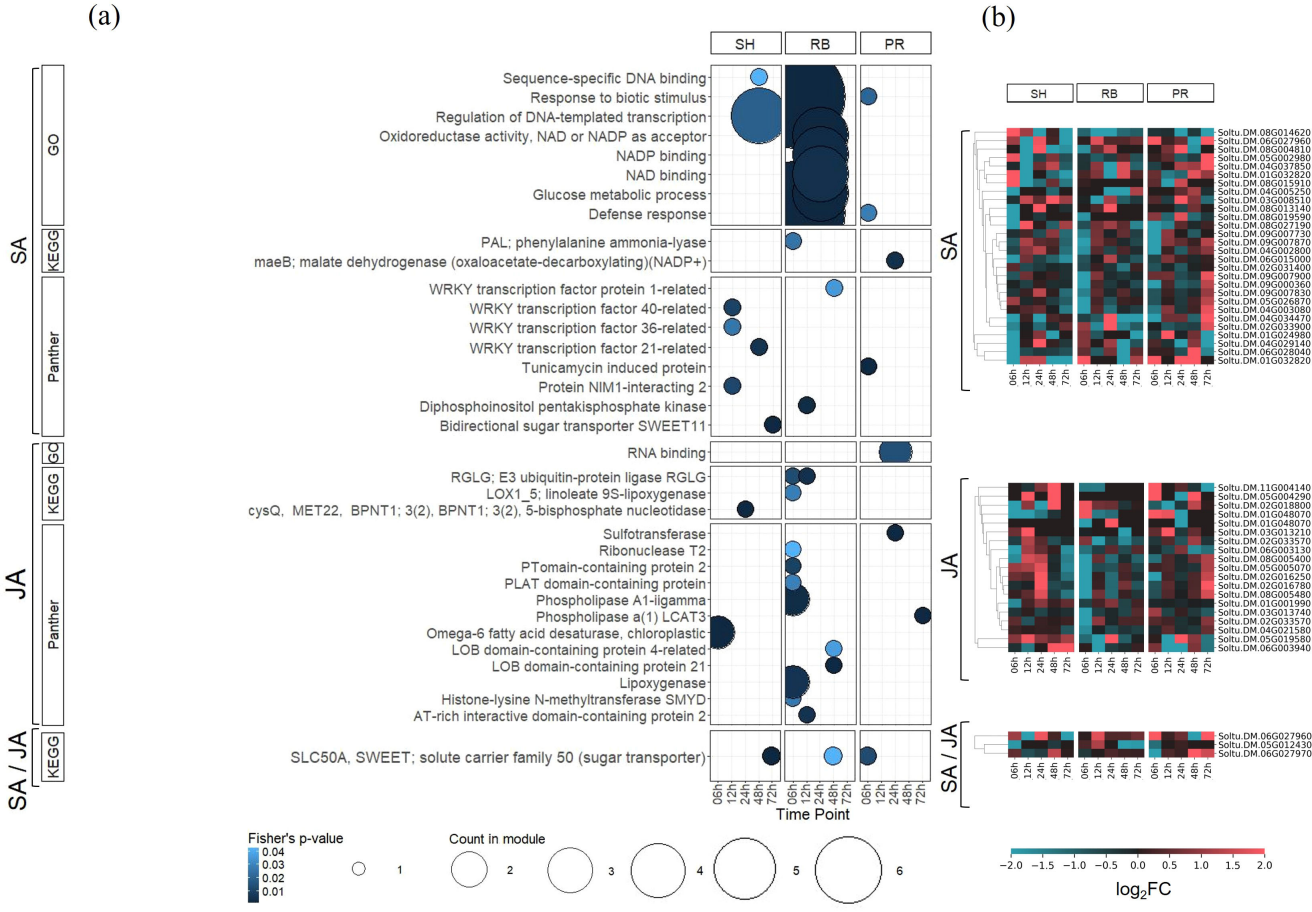
Functional enrichments of downregulated differentially expressed genes (DEGs) associated with PMTV infection in phytohormone-related pathways. **(a)** Bubble plots showing significantly enriched, upregulated functional terms from Gene Ontology (GO), Kyoto Encyclopedia of Genes and Genomes (KEGG), and PANTHER ontology (Panther) analyses. **(b)** Heatmaps showing expression patterns of genes with each enriched functional category, identified as responsive to PMTV infection through differential gene expression (DGE) analysis. The x-axis indicates the time points of sample collection post-pathogen inoculation across different potato cultivars. SA, salicylic acid; JA, jasmonic acid; SH, Shepody; RB, Russet Burbank; PR, Premier Russet.

Comparisons between non-viruliferous *Sss*-inoculated and PMTV-carrying *Sss*-inoculated samples identified 1341 DEGs associated with PMTV infection (663 in SH, 527 in RB, and 450 in PR). Of these, 925 genes were upregulated (473 in SH, 283 in RB, and 333 in PR), while 641 genes were downregulated (261 in SH, 272 in RB, and 146 in PR). PMTV triggered prominent jasmonic acid (JA)-related responses, particularly in SH at early time points (**Figure 3**), whereas JA-associated categories were downregulated in RB at multiple time points (**Figure 4**). Other phytohormone-related functional terms did not show clear cultivar-associated enrichment patterns (**Supplementary Figures 24**, **S25**). Cell wall-associated terms were upregulated in both SH and PR, while other cell wall categories were downregulated in SH and RB (**Supplementary Figure 26**, **S27**), indicating complex structural remodeling during PMTV infection. Interestingly, defense-related terms (**Supplementary Figures 28**, **29**) were downregulated in SH at multiple time points, suggesting that this reprogramming is likely advantageous for PMTV infection.

### Gene co-expression network analysis of *Sss*-associated infection

**Figure 5:**
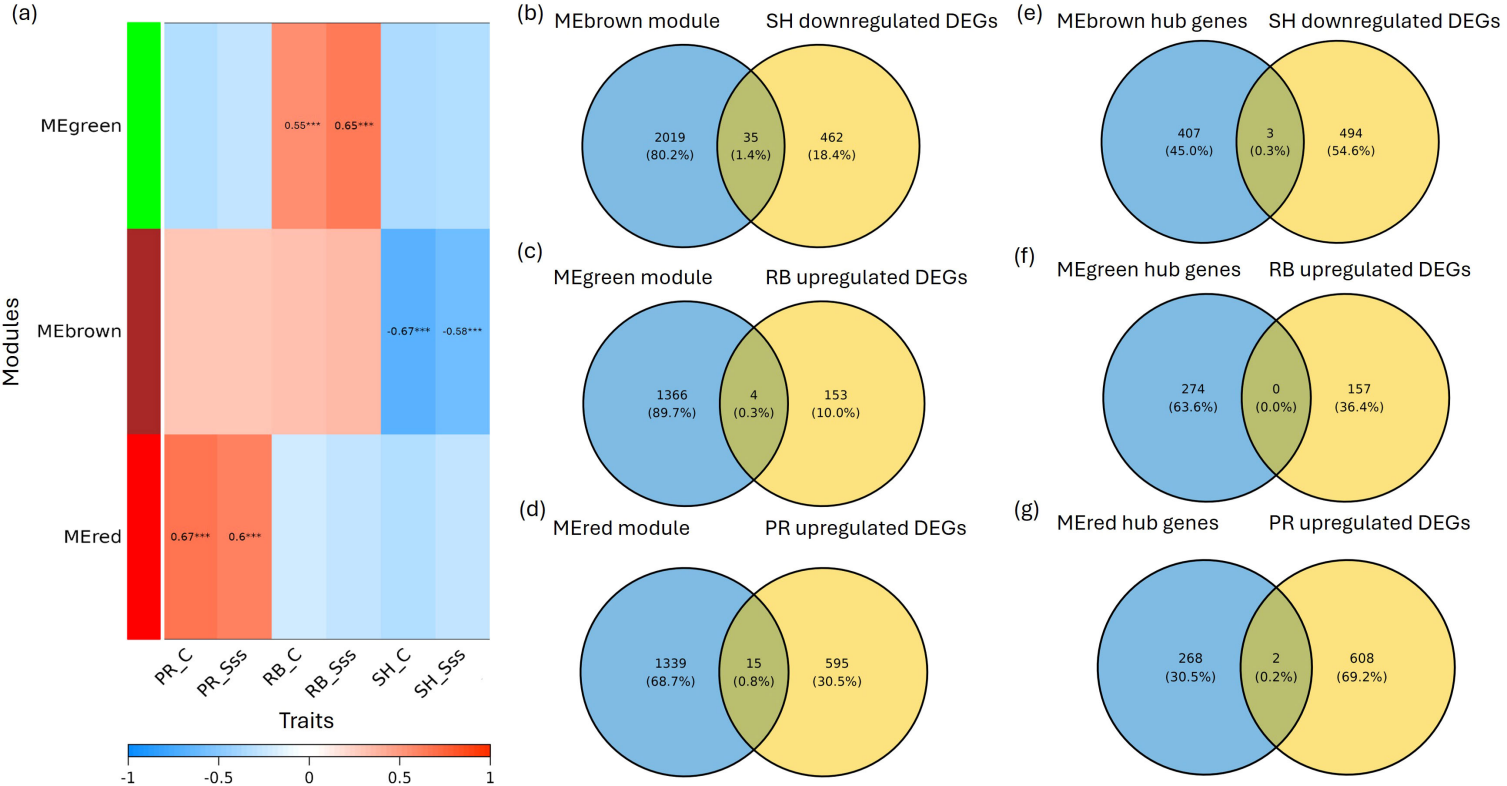
*Sss* infection-associated genes identified from Gene Co-expression Network (GCN) analysis and their overlap with differentially expressed genes (DEGs). **(a)** Heatmap showing significantly associated modules identified from GCNs constructed using transcriptomic data from control (denoted by “_C” following the cultivar acronym) and non-viruliferous *Sss-*inoculated samples (denoted by “_Sss”). Cultivar acronyms are SH (Shepody), RB (Russet Burbank), and PR (Premier Russet). Correlation coefficients are shown only for modules that exhibited a significant association with the trait. Asterisks indicate statistical significance with * for p< 0.05 ** for p< 0.01, and *** p < 0.001. A correlation coefficient threshold of 0.6 was applied for module selection, and the module most strongly associated with each cultivar was retained. The x-axis represents traits derived from sample metadata, and the y-axis indicates module names. **(b-g)** Venn diagrams showing the overlap between genes within each selected module or hub genes and DEGs. **(b, e)** show overlaps between downregulated DEGs and the module or hub associated with the SH cultivar. **(c, f)** show overlaps between upregulated DEGs and module or hub associated with the RB cultivar. **(d, g)** show overlaps between upregulated DEGs and module or hub associated with the PR cultivar. A total of 52 genes overlapping between significantly associated modules or hubs and the corresponding DEGs were listed in **Table 1**.

GCN analysis of mock-inoculated and non-viruliferous *Sss* samples identified 61 co-expression modules. Modules were assigned color identifiers, and module trait associations were evaluated across all metadata variables (**Supplementary Figure 30**). Modules with correlation > 0.6 and p < 0.05 were selected. Three trait-associated modules were identified (**Figure 5a**). The MEred module (1,353 genes) was strongly associated with PR under both mock- and *Sss*-treated conditions, suggesting constitutive and inducible transcriptional features linked to resistance. The MEgreen module (1,369 genes) was associated with RB specifically under *Sss-*treatment, reflecting infection-induced regulatory activity in the moderately resistant cultivar. In contrast, the MEbrown module (2,053 genes) was associated with SH under mock treatment, potentially indicating pre-existing transcriptional states that fail to be effectively remodeled upon infection in the susceptible background.

### Gene co-expression network analysis of PMTV-associated infection

**Figure 6:**
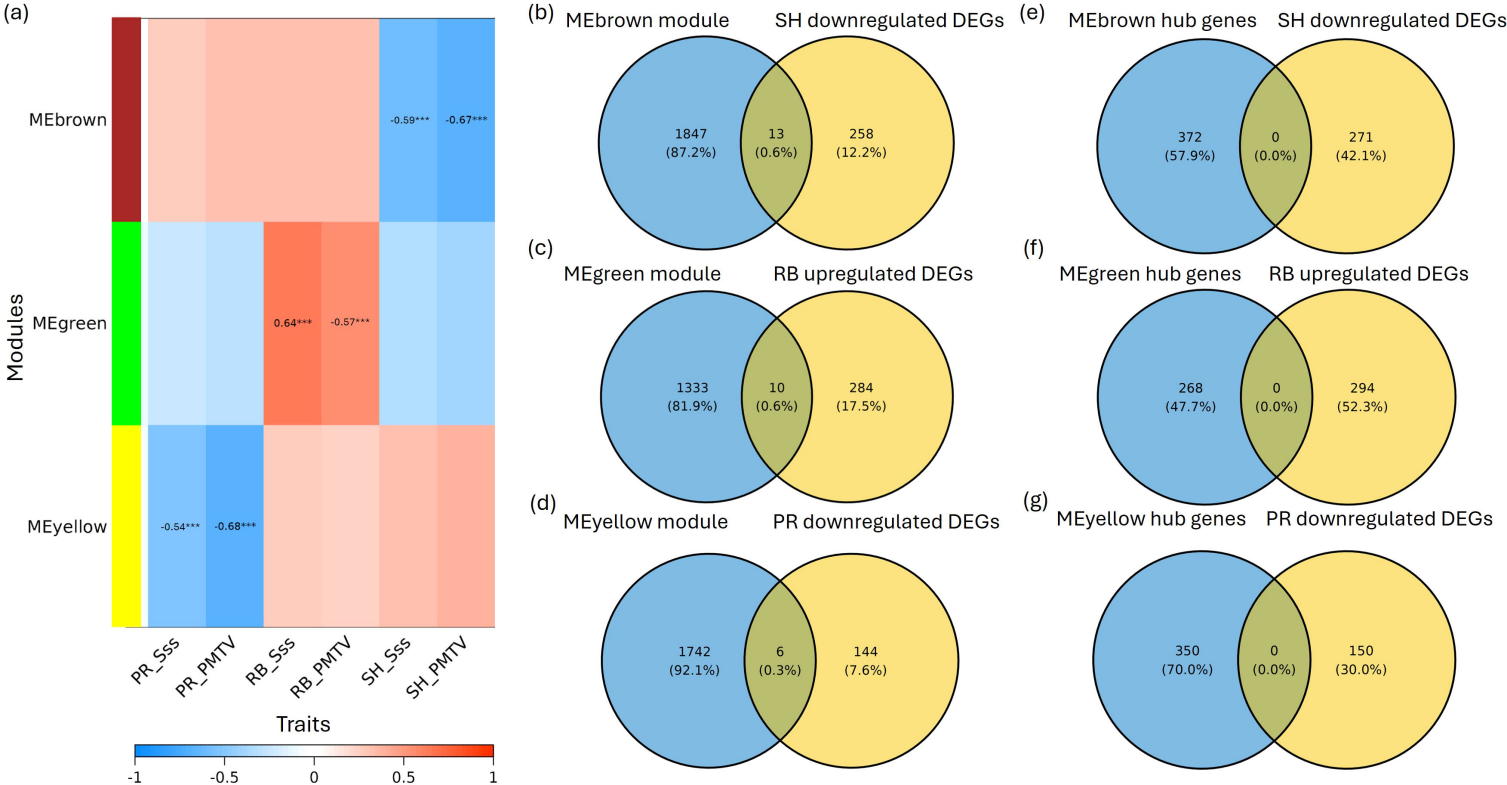
PMTV infection-associated genes identified from Gene Co-expression Network (GCN) analysis and their overlap with differentially expressed genes (DEGs). **(a)** Heatmap showing significantly associated modules identified from GCNs constructed using transcriptomic data from control (denoted by “_C” following the cultivar acronym) and non-viruliferous PMTV*-*inoculated samples (denoted by “_PMTV”). Cultivar acronyms are SH (Shepody), RB (Russet Burbank), and PR (Premier Russet). Correlation coefficients are shown only for modules that exhibited significant association with the trait. Asterisks indicate statistical significance with * for p< 0.05 ** for p< 0.01, and *** p < 0.001. A correlation coefficient threshold of 0.6 was applied for module selection, and the module most strongly associated with each cultivar was retained. The x-axis represents traits derived from sample metadata, and the y-axis indicates module names. **(b-g)** Venn diagrams showing the overlap between genes within each selected module or hub genes and DEGs. Panels **(b)** and **(e)** show overlaps between downregulated DEGs and module or hub associated with the SH cultivar. Panels **(c)** and **(f)** show overlaps between upregulated DEGs and module or hub associated with the RB cultivar. Panels **(d)** and **(g)** show overlaps between downregulated DEGs and module or hub associated with the PR cultivar. A total of 28 genes overlapping between significantly associated modules or hubs and the corresponding DEGs were listed in **Table 1**.

GCN analysis of non-viruliferous *Sss-*inoculated and PMTV-carrying *Sss-*inoculated samples identified 58 modules (**Supplementary Figure 31**). Using the same correlation thresholds (R^2^ > 0.6; p < 0.05), three modules showed significant trait associations (**Figure 6a**). The MEyellow module (1,654 genes) was associated with PR under viruliferous *Sss* treatment, indicating a coordinated transcriptional network likely contributing to PR-mediated tolerance or restriction of PMTV. The MEbrown module (1,752 genes) was associated with SH under viruliferous *Sss* treatment, reflecting extensive transcriptional reprogramming in the highly susceptible cultivar, potentially representing stress and susceptibility processes rather than effective defense. The MEgreen module (1,275 genes) was associated with RB only under non-viruliferous *Sss* treatment and was not associated with PMTV infection, suggesting that RB may fail to mount a coordinated PMTV-specific transcriptional response.

### Identification of candidate genes associated with *Sss* and PMTV infection using integrated DEG-GCN analyses

To identify genes associated with host responses to *Sss* infection, genes in each cultivar-associated GCN module were compared with corresponding DEGs (**Figures 5b-g**). This integrative approach enhances biological interpretation by capturing both transcriptional responsiveness. A total of 52 candidate genes were identified across all cultivars, including 35 in SH, 4 in RB, and 15 in PR (**Table 1**). The notable candidates included two glutathione-S-transferase (GST) genes, one downregulated in SH and the other upregulated in PR following *Sss* inoculation (**Table 1**), consistent with previous reports of GST involvement in *Sss* resistance (Balotf et al., 2025, 2022). Additionally, PR1-like and Bet v I (PR10) genes, typically associated with SA-mediated defense, were downregulated in SH.

**Table 1:** Fifty-two robust candidate genes associated with plant response to *Sss* infection.

Select module and cultivar DEG overlap	Gene	Gene name
Module Brown vs SH DEGs	Soltu.DM.01G014460.1	Feruloyl COA ortho-hydroxylase 1-related
	Soltu.DM.01G015840.5	Ring-type E3 ubiquitin transferase
	Soltu.DM.01G025140.2	Agenet-like domain, plant type-related
	Soltu.DM.01G029160.3	RNA polymerase subunit RPB8 (RPABC3/POLR2H)
	Soltu.DM.01G034630.3	Mitochondrial carrier protein MTM1-like isoform X1
	Soltu.DM.01G035220.1	Expressed protein
	Soltu.DM.01G046040.1	Peptidase inhibitor 16
	Soltu.DM.02G033450.5	β-1,4-mannosyl-glycoprotein GlcNAc transferase
	Soltu.DM.03G011810.2	LRR-RLK (RKF3)
	Soltu.DM.04G002760.1	PR10/Pathogenesis-related protein Bet v i family (Bet_v_1)
	Soltu.DM.04G035310.2	2-hydroxyacid dehydrogenase
	Soltu.DM.05G011990.1	F17l21.9
	Soltu.DM.05G026170.1	Protein cup-shaped cotyledon 3
	Soltu.DM.05G026510.2	FMN hydrolase (phosphatase)
	Soltu.DM.06G000540.1	Glutathione S-transferase (GST)
	Soltu.DM.06G016680.4	Terpene synthase C-terminal metal-binding domain
	Soltu.DM.08G014910.1	Root cap
	Soltu.DM.08G022440.1	Basic 7S globulin-like
	Soltu.DM.09G008220.2	CRM domain-containing protein
	Soltu.DM.09G024180.2	Linoleate 9s-lipoxygenase 5 (LOX5)
	Soltu.DM.09G024180.3	Linoleate 9s-lipoxygenase 5 (LOX5)
	Soltu.DM.09G025460.2	GDP-mannose 3,5-epimerase 1
	Soltu.DM.09G031540.1	WD_repeats_region domain-containing protein
	Soltu.DM.11G001840.2	TIR-containing NLR (TNL)
	Soltu.DM.11G002170.2	Non-canonical NLR
	Soltu.DM.11G009690.1	Metal transporter NRAMP5
	Soltu.DM.11G010240.2	Protein detoxification 54
	Soltu.DM.11G022110.3	LRR-RLP
	Soltu.DM.01G033730.1	Anion transporter 2, chloroplastic-related
	Soltu.DM.01G035880.2	DNAj homolog subfamily c member 2 (DNAJC2)
	Soltu.DM.01G037540.1	WD repeat-containing protein 48 (WDR48, UAF1)
	Soltu.DM.01G048740.1	PI-3-kinase-related kinase SMG-1 (SMG1)
	Soltu.DM.01G051600.6	Activator of basal transcription 1
	Soltu.DM.02G033450.5	N-acetylglucosaminyltransferase III (GnT-III)
	Soltu.DM.06G023650.3	Lysophospholipase II (LYPLA2)
Module Red vs PR DEGs	Soltu.DM.01G023360.2	Inosine/uridine-preferring nucleoside hydrolase
	Soltu.DM.01G029100.3	Glutathione S-transferase (GST)
	Soltu.DM.01G035200.2	Auxin response factor
	Soltu.DM.02G030250.2	Protein NSP-interacting kinase 1
	Soltu.DM.03G019160.5	Glutamate decarboxylase
	Soltu.DM.03G021350.3	EF-hand domain/Endonuclease/exonuclease/phosphatase
	Soltu.DM.03G025300.1	Indole-3-glycerol-phosphate synthase
	Soltu.DM.04G027270.1	Abscisic stress-ripening protein 1
	Soltu.DM.04G029110.2	F-box domain-containing protein
	Soltu.DM.09G023990.1	2-hydroxyisoflavanone dehydratase-like
	Soltu.DM.11G002510.2	Variant surface glycoprotein C-terminal domain
	Soltu.DM.11G010800.2	O-fucosyltransferase-like protein
	Soltu.DM.01G048740.4	PI-3-kinase-related kinase SMG-1 (SMG1)
	Soltu.DM.02G017990.2	RNA polymerase-associated protein RTF1 (RTF1)
	Soltu.DM.11G007900.2	F7O18.3 protein
Module Green vs RB DEGs	Soltu.DM.01G023450.3	Histidine-containing phosphotransfer protein 1
	Soltu.DM.02G030950.1	RRM domain-containing protein
	Soltu.DM.04G006170.2	Alpha-glucosidase
	Soltu.DM.05G008970.3	Pantothenate kinase/pantothenic acid kinase

Genes were identified through integrative analysis combining differential gene expression (DGE) and gene co-expression network (GCN) analyses for each cultivar, using genes from modules associated with the same corresponding cultivar (see **Figure 5**). SH, Shepody; RB, Russet Burbank; PR, Premier Russet.

A similar DEG-GCN integration approach applied to PMTV-associated transcriptomes (**Figure 6b-g**) identified 28 candidates (with 13 in SH, 10 in RB, and 6 in PR) (**Table 2**). These included GSTs and several genes of unknown function (**Table 2**).

**Table 2:** Twenty-eight robust candidate genes associated with plant response to PMTV infection.

Select module and cultivar DEG overlap	Gene	Gene name
Module Brown vs SH DEGs	Soltu.DM.01G032820.1	C2 calcium_lipid-binding endonuclease, phosphatase
	Soltu.DM.02G004910.1	3-hydroxy-3-methylglutaryl-coenzyme a reductase
	Soltu.DM.04G005020.2	UPFf0481 protein
	Soltu.DM.04G005020.4	UPF0481 protein
	Soltu.DM.04G034550.5	TF-B3 domain-containing protein
	Soltu.DM.06G004980.4	TIR-containing NLR (TNL)
	Soltu.DM.06G010770.1	Peroxidase
	Soltu.DM.06G017120.2	α-humulene/β-caryophyllene/germacrene D synthase (ZSS1)
	Soltu.DM.01G033730.2	Anion transporter 2, chloroplastic-related
	Soltu.DM.01G039520.8	Splicing factor, arginine/serine-rich 1/9 (SFRS1_9)
	Soltu.DM.05G004680.2	Protein of unknown function (DUF3326)
	Soltu.DM.06G022170.2	Ubiquitin-conjugating enzyme E2 J2
	Soltu.DM.08G021880.2	ESCRT-II complex subunit VPS25 (VPS25, EAP20)
Module Yellow vs PR DEGs	Soltu.DM.01G029100.6	Glutathione S-transferase (GST)
	Soltu.DM.03G023160.2	S-type anion channel SLAH2-like
	Soltu.DM.06G020210.1	Adenylate kinase
	Soltu.DM.06G033210.2	Frigida-like protein
	Soltu.DM.08G021330.2	Methyltransferase PMT21-related
	Soltu.DM.01G045850.3	Protein of unknown function
Module Green vs RB DEGs	Soltu.DM.01G026730.3	Choline transporter-like protein
	Soltu.DM.02G016120.2	DUF538 family protein
	Soltu.DM.05G008970.1	Pantothenate kinase/pantothenic acid kinase
	Soltu.DM.06G032710.2	RNA-directed DNA methylation 4
	Soltu.DM.08G023830.2	ATP-dependent Clp protease subunit 4 (chloroplast)
	Soltu.DM.11G008090.2	NAC domain-containing protein
	Soltu.DM.11G024460.2	Aldehyde oxidase/xanthine dehydrogenase FAD, [2Fe–2S]
	Soltu.DM.05G012830.2	Vacuole morphology and inheritance protein 14
	Soltu.DM.08G005440.4	Linoleate 9S-lipoxygenase (LOX1_5)
	Soltu.DM.09G005250.3	ACT domain-containing protein

Genes were identified through integrative analysis combining differential gene expression (DGE) and gene co-expression network (GCN) analyses for each cultivar, using genes from modules associated with the same corresponding cultivar (see **Figure 5**). SH, Shepody; RB, Russet Burbank; PR, Premier Russet.

### Immune receptor genes associated with *Sss* and PMTV infection

Plant immunity is primarily mediated by pattern-triggered immunity (PTI) and effector-triggered immunity (ETI) (Jones and Dangl, 2006). PTI is activated by pattern recognition receptors (PRRs), most commonly LRR-containing receptor-like kinases (LRR-RLKs) and receptor-like protein (LRR-RLP), while ETI is mediated by nucleotide-binding leucine-rich repeat (NLR) receptors. Because these receptors are central to plant immunity, we specifically examined whether they were represented among our narrowed candidate genes.

**Figure 7:**
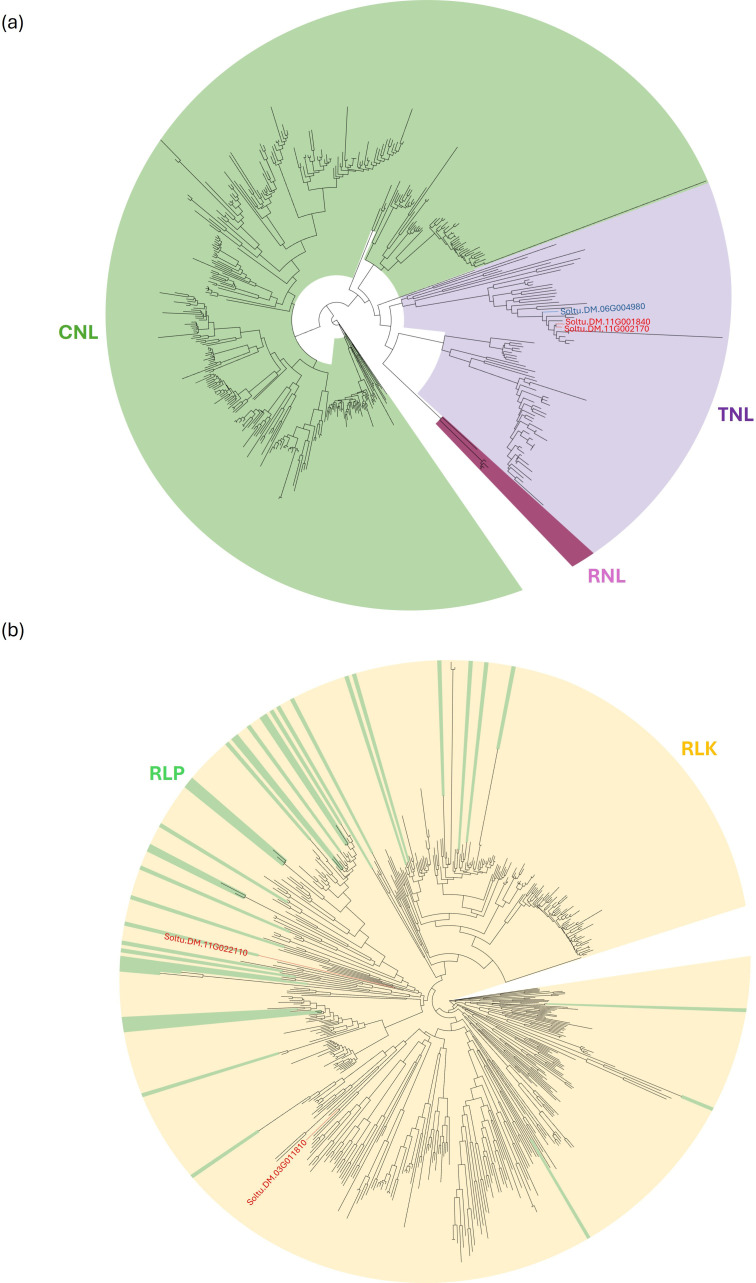
Plant immune receptors identified from the 80-gene list (**Tables 1**, **2**) associated with *Sss* or PMTV infection. **(a)** Phylogenetic tree of 438 potato NLRs constructed using protein sequences containing NB-ARC and LRR domains, identified through keyword searches in the Phytozome online genomic resource (*Solanum tuberosum* v6.1). CNL, coiled-coil NLR; RNL, RPW8-NLR; TNL, Toll/interleukin-1 receptor NLR. **(b)** Phylogenetic tree of 529 potato RLKs and RLPs. RLKs were identified via keyword searches for genes containing both LRR and kinase domains, while RLPs were identified using the keyword “receptor-like proteins” in the Phytozome. Domain annotation of all candidate genes was performed using InterProScan, with genes containing kinase domains classified as RLKs and those lacking kinase domains classified as RLPs. Genes highlighted in red are associated with responses to *Sss* infection, while the gene highlighted in blue is associated with PMTV infection. Phylogenetic trees were generated using NGPhylogeny.fr with MAFFT alignment and the FastTree maximum likelihood method.

Among the 52 *Sss-*associated candidate genes, four encoded LRR-containing proteins, including two NLR genes (Soltu.DM.11G001840.2, Soltu.DM.11G002170.2), one LRR-RLK gene (Soltu.DM.03G011810.2) and one LRR-RLP gene (Soltu.DM.11G022110.3) (**Figure 7**; **Table 1**). Both NLR and LRR-RLK/RLP were associated with SH and were downregulated following *Sss* infection, suggesting suppression of immune signaling in the susceptible cultivar. Among them, one NLR gene Soltu.DM.11G002170.2 lacked the canonical NB-ARC domain but may still contribute to the regulation of defense responses.

Among the 28 PMTV-associated candidate genes, one NLR-encoding gene Soltu.DM.06G004980.4 was found (**Figure 7a**; **Table 2**), and showed reduced expression in SH following PMTV infection.

Interestingly, the three NLRs identified (Soltu.DM.11G001840 and Soltu.DM.11G002170 associated with *Sss* infection and Soltu.DM.06G004980 associated with PMTV infection) are closely related, sharing 53–67% identity and 63–73% similarity.

### Co-expression core-modules of candidate PRRs and NLRs associated with *Sss* and PMTV infection

**Figure 8:**
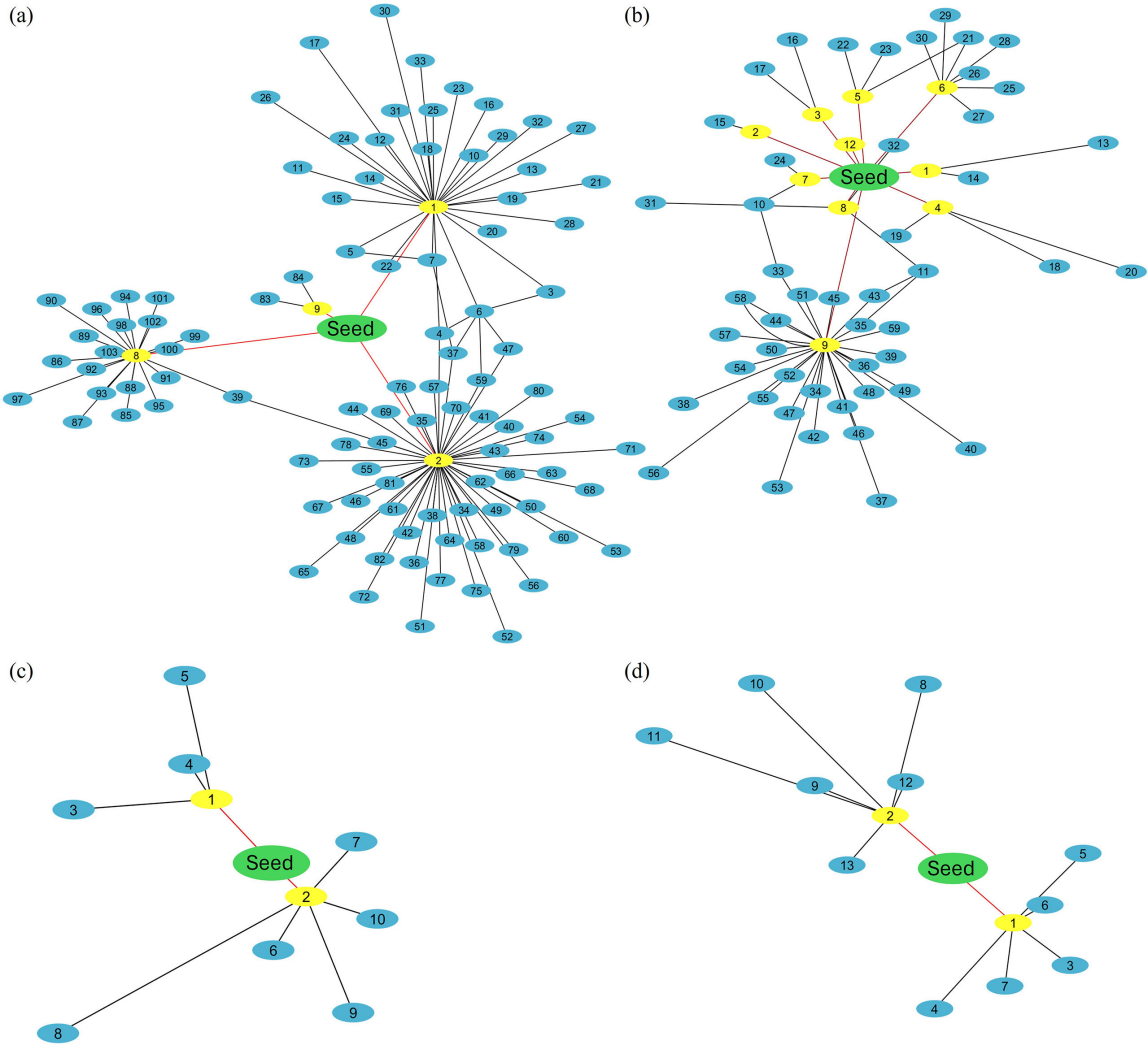
Co-expression core-modules of NLR and PRR candidate genes identified in this study. Non- NLR genes Soltu.DM.11G001840.2 **(a)** and Soltu.DM.11G002170.2 **(b)**, and the LRR-RLK gene (putative PRR) Soltu.DM.03G011810.2 **(c)**, all associated with *Sss* infection, as well as the NLR gene Soltu.DM.06G004980.4 **(d)** associated with PMTV infection (see **Figure 7**), are shown as seed nodes (green). First- and second-degree neighbors are indicated in yellow and blue, respectively. Edges connecting candidate genes to their first-degree neighbors are shown in red. Genes corresponding to the numbered nodes in each core-module are listed in **Table 3**. Modules were visualized using Cytoscape software (Shannon et al., 2003).

To further explore coordinated defense responses, we examined core-modules associated with candidate PRRs and NLRs. First- and second-neighbor core-modules were extracted from corresponding GCN modules using a correlation cutoff of R^2^ ≥ 0.28. These core-modules represent co-expressed genes that may participate in cultivar-specific resistance or susceptibility (**Figure 6**). Genes common to both core-modules and DEGs were also identified to refine candidate lists (**Supplementary Table 2**). For *Sss-*associated NLR Soltu.DM.11G001840.2, a potential RLK is present as a first neighbor, and additional RLKs, TIR-containing NLRs (TNLs), and a regulatory defense RIN4-like protein appeared as second neighbors (**Figure 8a**, **Table 3**). One of these TNLs, Soltu.DM.11G001840.3, shows upregulation in SH at 24 h post-inoculation. The *Sss*-associated non-canonical NLR Soltu.DM.11G002170.2 was linked to two potential receptor-like kinases as second neighbors (**Figure 8b**). The core-module of *Sss*-associated LRR-RLK Soltu.DM.03G011810.2, a coild-coil NLR (CNL; Soltu.DM.06G022870.2) is assicited as the first neighbor (**Figure 8c**, **Table 3**). For PMTV-associated NLR gene Soltu.DM.06G004980.4, the core-module included Bet v I protein which is also known as PR10 (**Figure 8d**, **Table 3**). In DEGs, this NLR was observed as upregulated in PR at 72 h but downregulated in SH at the same time point. (**Supplementary Table 2**).

**Table 3:** Gene IDs and names of core-module members associated with NLR and PRR candidate genes responsive to *Sss* and PMTV infection.

Network	Ref No	Gene	Gene_name
**Figure 8a**	Seed	Soltu.DM.11G001840.2	TIR-containing NLR (TNL)
	1	Soltu.DM.11G019890.1	Gene of unknown function
	2*	Soltu.DM.01G038640.1	LRR-RLK
	3	Soltu.DM.09G022120.4	Myb DNA-binding/Myb_CC_LHEQLE
	4*	Soltu.DM.11G001490.1	THAP domain-containing protein 4
	5	Soltu.DM.11G019760.2	ADP-ribosyl cyclase_cyclic adp-ribose hydrolase, TIR
	6	Soltu.DM.11G002780.1	K homology domain-containing protein
	7	Soltu.DM.01G040040.3	Exonuclease mut-7 homolog
	8	Soltu.DM.02G012020.1	Cathepsin b-like
	9	Soltu.DM.01G045530.1	Heat shock protein
	10	Soltu.DM.09G020280.1	Protein kinase domain-containing protein
	11	Soltu.DM.09G022740.1	OS09G0392200 protein
	12	Soltu.DM.01G002090.1	S-locus glycoprotein/Tyrosine kinase/GM2-AP
	13	Soltu.DM.09G025370.1	Cytochrome p450 cyp72a219-like
	14	Soltu.DM.09G025410.1	Gene of unknown function
	15	Soltu.DM.09G025940.1	Inhibitor of trypsin and hageman factor-like
	16	Soltu.DM.09G027490.1	DYW_deaminase domain-containing protein
	17	Soltu.DM.09G027580.1	Protein transport protein SEC20 (SEC20)
	18	Soltu.DM.01G002170.1	Spirosolane 16-oxygenase
	19	Soltu.DM.09G030610.1	Tetratricopeptide repeat (TPR)/Glyco_transf_41
	20	Soltu.DM.09G030960.1	Steroid 17alpha-monooxygenase (CYP17A)
	21	Soltu.DM.11G001480.1	Auxin-responsive protein SAUR65-like
	22	Soltu.DM.01G002210.1	Spirosolane 16-oxygenase
	23	Soltu.DM.11G001700.1	Protein f25b4.8, isoform a
	24	Soltu.DM.11G001840.3	TIR-containing NLR (TNL)
	25	Soltu.DM.11G002220.1	Enoyl reductase (ER) domain-containing protein
	26	Soltu.DM.11G002290.1	Galactose-binding protein-related
	27	Soltu.DM.11G003140.1	Mitochondrial dicarboxylate carrier
	28	Soltu.DM.11G003680.1	Protein kinase domain-containing protein
	29*	Soltu.DM.11G003830.1	Type II inositol polyphosphate 5-phosphatase 15
	30	Soltu.DM.11G006250.1	MFS domain-containing protein
	31	Soltu.DM.11G009980.1	LRR-RLK (EMS1)
	32	Soltu.DM.11G012390.1	Gene of unknown function
	33	Soltu.DM.11G012920.1	GDSL esterase_lipase 2-like
	34	Soltu.DM.09G006380.1	Ubiquitin-protein ligase
	35	Soltu.DM.09G007090.3	Uncharacterized protein (K06940)
	36	Soltu.DM.01G030820.1	Stizolobate synthase
	37	Soltu.DM.09G009300.1	PRA1 family protein
	38*	Soltu.DM.09G009610.1	Neurolysin/neurotensin endopeptidase.
	39	Soltu.DM.09G009870.1	SWIM-type domain-containing protein
	40	Soltu.DM.09G010040.1	Gene of unknown function
	41	Soltu.DM.01G030880.1	E3 ubiquitin-protein ligase RNF25 (RNF25, AO7)
	42	Soltu.DM.09G010610.1	Proline-rich receptor-like protein kinase PERK8
	43	Soltu.DM.01G030940.2	Integrator complex subunit 4 (INTS4)
	44	Soltu.DM.09G011400.1	Gene of unknown function
	45	Soltu.DM.09G013060.1	Josephin [EC:3.4.19.12] (JOSD)
	46	Soltu.DM.09G013670.1	OS08G0464400 PROTEIN
	47	Soltu.DM.09G015240.1	Protein of unknown function (DUF707)
	48	Soltu.DM.09G016370.1	Gene of unknown function
	49	Soltu.DM.09G016750.1	Domon domain-containing protein
	50	Soltu.DM.09G019110.2	Ribulose bisphosphate carboxylase large chain
	51	Soltu.DM.09G019390.2	Trans-2,3-dihydro-3-hydroxyanthranilate isomerase
	52	Soltu.DM.09G019710.1	Sarcalumenin
	53	Soltu.DM.01G031350.2	Malate dehydrogenase 3, cytoplasmic
	54	Soltu.DM.09G020670.1	Replication protein A interacting protein
	55	Soltu.DM.09G022190.3	Set domain-containing protein
	56	Soltu.DM.09G022290.1	MFS domain-containing protein
	57	Soltu.DM.09G023300.1	Rubber elongation factor protein (REF) (REF)
	58	Soltu.DM.09G023640.1	Ring-h2 finger protein atl16
	59	Soltu.DM.09G027060.1	Jacalin-type lectin domain-containing protein
	60	Soltu.DM.09G027170.1	SAM-dependent methyltransferase
	61	Soltu.DM.11G001630.1	Potassium channel
	62	Soltu.DM.11G002070.1	TMV resistance protein N (TIR protein)
	63	Soltu.DM.11G002150.1	TIR-containing NLR (TNL)
	64	Soltu.DM.11G002670.1	Mannitol dehydrogenase-related
	65	Soltu.DM.11G003080.1	Mannitol dehydrogenase-related
	66	Soltu.DM.11G005370.1	Gene of unknown function
	67	Soltu.DM.11G007360.1	Nuclear transport factor 2 (NTF2) family protein
	68	Soltu.DM.11G007610.1	Calcium-dependent protein kinase 3
	69	Soltu.DM.01G033260.5	acetyl-CoA carboxylase α-subunit (accA)
	70	Soltu.DM.01G033300.1	Glycosyltransferase
	71	Soltu.DM.11G012170.1	MDIS1-interacting RLK2/HSL1-like isoform X2
	72	Soltu.DM.11G012500.1	Proteinase inhibitor type-2 TR8
	73	Soltu.DM.11G023920.3	D-amino-acid transaminase, chloroplastic
	74	Soltu.DM.11G024510.2	Pentatricopeptide repeat-containing protein gun1
	75	Soltu.DM.11G024650.1	Succinate dehydrogenase/succinic dehydrogenase
	76	Soltu.DM.01G034410.1	BAG family molecular chaperone regulator 6
	77	Soltu.DM.11G026370.4	NOT transcription complex subunit VIP2 isoform X1
	78	Soltu.DM.01G034630.3	Mitochondrial carrier protein MTM1-like isoform X1
	79	Soltu.DM.01G036710.1	Annexin
	80	Soltu.DM.01G037100.1	Protein of unknown function (DUF1517)
	81	Soltu.DM.01G037540.1	WD repeat-containing protein 48 (WDR48, UAF1)
	82	Soltu.DM.01G037960.1	Sugar-porter family protein 5
	83	Soltu.DM.09G017260.4	RPM1-interacting protein 4 (RIN4)-like isoform x1
	84	Soltu.DM.01G031200.4	Dnaj protein homolog 2-like isoform x1
	85	Soltu.DM.01G029100.2	Glutathione S-transferase (GST)
	86	Soltu.DM.08G023290.1	Helicase atp-binding domain-containing protein
	87	Soltu.DM.08G023400.2	Poly(U)-specific endoribonuclease (ENDOU, PP11)
	88	Soltu.DM.08G023560.1	RLK family protein
	89	Soltu.DM.08G023760.1	Deoxyhypusine monooxygenase/DOHH
	90	Soltu.DM.09G001760.2	BHLH domain-containing protein
	91	Soltu.DM.09G006030.1	Serine carboxypeptidase-like 50
	92	Soltu.DM.09G018470.2	MADS-box domain-containing protein
	93	Soltu.DM.09G021950.1	Glutamine synthetase, chloroplastic_mitochondrial
	94	Soltu.DM.09G022750.1	Cytosolic iron-sulfur protein assembly protein CIAO1
	95	Soltu.DM.09G031080.1	Aldo-keto reductase 2-related
	96	Soltu.DM.11G010930.1	Scarecrow-like protein 26
	97	Soltu.DM.11G012720.1	Mitochondrial transcription termination factor
	98	Soltu.DM.11G015100.1	Ethylene-responsive transcription factor LEP-like
	99	Soltu.DM.01G036570.1	Gene of unknown function
	100	Soltu.DM.01G002660.1	RNA-binding protein CP33, chloroplastic
	101	Soltu.DM.02G003610.2	Urease accessory protein (URED, UREH)
	102	Soltu.DM.02G003720.2	ADP-ribosyl cyclase_cyclic ADP-ribose hydrolase, TIR
	103	Soltu.DM.02G010800.1	28s ribosomal protein s21, mitochondrial
**Figure 8b**	Seed	Soltu.DM.11G002170.1	Non-canonical NLR
	1	Soltu.DM.06G023380.1	F-box domain-containing protein
	2	Soltu.DM.06G034520.1	PPM-type phosphatase domain-containing protein
	3	Soltu.DM.01G027380.1	Carotenoid 9,10(9’,10’)-cleavage dioxygenase 1
	4	Soltu.DM.08G016760.1	RAS suppressor/LRR protein in muscle apoptosis
	5	Soltu.DM.08G018410.1	Peroxidase
	6	Soltu.DM.09G019550.1	Protein trichome birefringence-like 11
	7*	Soltu.DM.09G009610.1	Neurolysin/neurotensin endopeptidase
	8	Soltu.DM.11G002360.2	F-box domain-containing protein
	9	Soltu.DM.11G020720.2	Protein nuclear fusion defective 4-like
	10*	Soltu.DM.01G038640.1	LRR-RLK
	11	Soltu.DM.02G005450.4	Transformer-2 protein (TRA2)
	12	Soltu.DM.06G021570.1	SFBBbeta protein
	13	Soltu.DM.11G019920.1	ABC-type xenobiotic transporter
	14	Soltu.DM.02G018140.1	Receptor-like serine_threonine-protein kinase
	15	Soltu.DM.01G041630.1	MEP cytidylyltransferase.
	16	Soltu.DM.04G014290.1	Protein NSP-interacting kinase 3
	17	Soltu.DM.04G032590.1	Protein NPGR1
	18	Soltu.DM.01G029820.1	Gene of unknown function
	19*	Soltu.DM.11G003830.1	Type II inositol polyphosphate 5-phosphatase 15
	20	Soltu.DM.02G029010.1	Membrane protein
	21	Soltu.DM.08G005450.1	Gene of unknown function
	22	Soltu.DM.01G031780.1	Gene of unknown function
	23	Soltu.DM.11G000730.2	DNA-directed RNA polymerase I & III subunit RPAC2
	24	Soltu.DM.02G012680.1	Fad-binding PCMH-type domain-containing protein
	25	Soltu.DM.08G017070.1	ZFYVE26 (FYVE zinc finger protein 26)
	26	Soltu.DM.08G018000.1	N-acetyltransferase domain-containing protein
	27	Soltu.DM.08G019750.1	Glycosyltransferase
	28	Soltu.DM.01G029670.1	Ring-h2 finger protein atl3-like
	29	Soltu.DM.09G000550.1	Transcription factor divaricata-like
	30	Soltu.DM.09G017690.1	Ring-type domain-containing protein
	31	Soltu.DM.08G025930.1	Class I peptide chain release factor
	32	Soltu.DM.03G005210.1	Tubulin, ftsz, gtpase domain
	33*	Soltu.DM.11G001490.1	THAP domain-containing protein 4
	34	Soltu.DM.11G003160.4	Protein curvature thylakoid 1d, chloroplastic
	35	Soltu.DM.11G005920.1	Glycosyltransferase
	36	Soltu.DM.11G005960.1	Glycosyltransferase
	37	Soltu.DM.01G032850.1	Abieta-7,13-dien-18-ol hydroxylase./cyp720b1.
	38	Soltu.DM.11G006690.1	F-box protein pp2-b12-related
	39	Soltu.DM.01G033020.1	Abieta-7,13-dien-18-ol hydroxylase./cyp720b1.
	40	Soltu.DM.11G007780.1	AT-hook motif nuclear-localized protein 14
	41	Soltu.DM.11G008840.1	WAT1-related protein
	42	Soltu.DM.11G009190.1	RNAse H domain-containing protein
	44	Soltu.DM.11G013590.1	Partial AB-hydrolase lipase domain
	45	Soltu.DM.11G014810.1	LD22649P
	46	Soltu.DM.11G017310.1	Protein T08G11.1, isoform a
	47	Soltu.DM.01G033690.1	U-box domain-containing protein 33
	48	Soltu.DM.11G017490.3	MADS-box domain-containing protein
	49	Soltu.DM.11G017920.1	Endo-polygalacturonase/polygalacturonase
	50	Soltu.DM.11G018000.1	Zinc finger A20/AN1 stress-associated protein 8-like
	51	Soltu.DM.11G018030.2	B3 domain-containing protein REM10-like
	52	Soltu.DM.11G018510.1	RNA polymerase-associated protein RTF1 homolog
	53	Soltu.DM.11G018570.1	Gene of unknown function
	54	Soltu.DM.11G018650.2	Protein kinase 2b, chloroplastic-like
	55	Soltu.DM.11G019150.2	Gene of unknown function
	56	Soltu.DM.11G019370.1	DNAJ/Tetratricopeptide repeat protein
	57	Soltu.DM.11G019730.1	Subtilisin-like protease sbt1.9
	58	Soltu.DM.11G020010.1	Thymidine kinase, cytosolic
	59	Soltu.DM.11G020310.1	OS10G0100500 protein
**Figure 8c**	Seed	Soltu.DM.03G011810.2	LRR-RLK (RKF3-related)
	1	Soltu.DM.06G022870.2	Coiled-coil NLR (CNL)
	2	Soltu.DM.02G006820.1	BES1/BZR1 homolog protein 2
	3	Soltu.DM.04G006470.1	OS01G0859400 protein
	4	Soltu.DM.04G020150.1	Gene of unknown function
	5	Soltu.DM.04G022940.1	Transmembrane protein
	6	Soltu.DM.03G018630.1	Cysteine protease inhibitor 1
	7	Soltu.DM.03G019730.1	ARM repeat superfamily protein
	8	Soltu.DM.03G021420.1	Gene of unknown function
	9	Soltu.DM.04G006660.1	Diboa-glucoside dioxygenase BX6
	10	Soltu.DM.04G011800.1	Phosphatidylinositol glycan, class Q (PIGQ, GPI1)
**Figure 8d**	Seed	Soltu.DM.06G004980.4	TIR-containing NLR (TNL)
	1	Soltu.DM.02G020880.2	RAS-related protein RAB-5C
	2	Soltu.DM.02G022470.1	Protein-serine_threonine phosphatase
	3	Soltu.DM.04G032220.3	Growth-regulating factor 6
	4	Soltu.DM.06G016320.1	(-)-germacrene D synthase
	5	Soltu.DM.06G017540.1	Transmembrane EMP24 domain-containing protein 10
	7	Soltu.DM.08G023060.1	Methyltransferase PMT14-related
	8	Soltu.DM.04G030140.1	Alcohol dehydrogenase-like 2
	9	Soltu.DM.08G024220.1	Ring-type E3 ubiquitin transferase
	10	Soltu.DM.09G000800.1	F-box associated domain (FBA_3)/F-box-like
	11	Soltu.DM.09G001660.1	Diacylglycerol kinase theta-like
	12	Soltu.DM.09G007670.1	Kirola-like, Bet_v_1
	13	Soltu.DM.11G009170.1	SRR1-like protein

The resentence numbers correspond to the positions of genes within the core-modules shown in **Figure 8**. Asterisk (*) indicates genes that overlap between the two modules.

## Discussion

Genetic resistance is among the most effective and sustainable strategies for protecting commercial crops from devastating pathogens. However, for powdery scab and PMTV-associated diseases, the limited understanding of the genetic basis of resistance has hindered progress toward deploying host resistance in potato production (Jayasinghe et al., 2026). In this study, we identified 80 candidate genes, including several encoding NLRs and PRRs from transcriptomic analyses, which may function as resistance determinants or molecular markers for breeding resistance against *Sss* and PMTV. Importantly, integration of DGE analysis with GCN analysis provided multiple layers of statistical support, strengthening confidence in the identified candidates. Below, we discuss key genes and pathways that may directly contribute to potato defense responses against *Sss* and PMTV.

### Involvement of SA in response to *Sss*

SA is a central regulator of plant immunity, particularly against biotrophic pathogens (Glazebrook, 2005). Our functional enrichment analyses revealed that SA-associated biological processes and pathways were downregulated in the susceptible cultivar SH following *Sss* infection, whereas these functions were upregulated in the resistant cultivar PR. This contrasting transcriptional regulation suggests that SA signaling plays an important role in mediating differential resistance responses to *Sss*. Downregulation of SA pathways in SH may therefore contribute to its susceptibility.

Consistent with these observations, SA-associated genes such as pathogenesis-related protein Bet v I family (PR10) were downregulated in SH and were induced among defense-related candidates identified by the combined DGE-GCN pipeline. Our results align with previous studies reporting the involvement of SA in *Sss* resistance, supporting our findings and reinforcing the role of phytohormone-mediated defenses following *Sss* infection (Jayasinghe et al., 2025; Lekota et al., 2019). Jayasinghe et al. (2025) further described the roles of NPR1 and NPR3, key SA receptors, in mediating the defense response to *Sss*. In addition, exogenous application of SA to roots prior to *Sss* infection reduced relative *Sss* levels, while SA accumulation increased in roots upon infection. Together, these findings suggest that targeting SA-mediated defense mechanisms may provide an effective strategy for managing powdery scab disease.

### Auxin as a potential regulator of powdery scab disease symptoms

The role of auxin in plant defense regulation has been characterized by numerous studies. In general, auxin signaling has been reported to enhance resistance to necrotrophic pathogens while promoting susceptibility to biotrophic pathogens (Kunkel and Joshua, 2021; Kazan and Lyons, 2014). In this study, we observed that two auxin-related genes, auxin response factor (ARF) and indole-3-glycerol-phosphate synthase (IGPS), were both upregulated in PR following *Sss* infection (**Table 1**). Additionally, the Auxin-responsive protein SAUR65-like was observed within the core-network of an NLR gene that was downregulated in SH after infection (**Table 3**).

The involvement of auxin in *Sss*-associated disease regulation has also been reported previously. Our prior work showed that indole-3-acetic acid (IAA), the primary active form of auxin, accumulates during the early stages of *Sss* infection (Jayasinghe et al., 2025). Clarke et al. (2020) demonstrated that foliar application of low concentrations of the synthetic auxin 2,4-D reduced disease severity of powdery scab under field conditions. Together, these findings suggest a potential role for auxin in contributing to disease resistance responses.

In contrast, auxin has been implicated in the development of root gall symptoms in diseases caused by other pathogens, including root-knot nematodes and *Plasmodiophora brassicae* (Ando et al., 2023; Suzuki et al., 2022; Ludwig-Müller et al., 2009). Both pathogens induce gall-like structures similar to those observed in *Sss*, and suppression of auxin-related genes has been sown to reduce gall size (Päsold and Ludwig-Müller, 2013; Suzuki et al., 2022). Taken together, these observations suggest that elevated auxin levels may be associated with root gall development, while modulation or reduction of auxin signaling could potentially limit root gall formation and, consequently, sporosori development. However, further investigation is required to clarify the precise role of auxin in *Sss*-induced disease development and resistance.

### Potential immune receptors in defense against *Sss* and PMTV

Plant defense responses are regulated by complex mechanisms and can be categorized into two main types: PTI and ETI (Jones and Dangl, 2006). The regulation of these pathways determines whether a plant is susceptible or resistant to a given pathogen. PTI is activated by the recognition of pattern-associated molecular patterns (PAMPs) by cell surface-localized receptors known as PRRs. In contrast, ETI is triggered by the recognition of pathogen-derived effector proteins by intracellular receptors known as resistance (R) proteins or NLRs.

PRRs are typically classified as receptor-like kinase (RLK) or receptor-like protein (RLPs), depending on presence or absence of an intracellular kinase domain, respectively. Although PRRs possess diverse extracellular domains, this study focused on LRR-containing proteins, as they represent a major class within the RLK family. We identified one LRR-RLK (Soltu.DM.03G011810.2) one LRR-RLP (Soltu.DM.11G022110.3) genes as a potential PRR associated with *Sss* infection. The core-module associated with the LRR-RLK (Soltu.DM.03G011810.2) included a CNL gene (Soltu.DM.06G022870.2) (**Table 3**), which has been described as PSH-RGH7 in prior genome versions. This gene has been described as associated with root-knot nematode resistance (Lee et al., 2012). Thus, co-expression of these genes indicates a potentially important resistance module that warrants functional validation.

We also identified three NLR-encoding genes, two associated with *Sss* infection and one associated with PMTV infection. These were TIR-NLR (TNL), classified as sensor NLRs responsible for pathogen-derived effector recognition (Liu et al., 2023; Maruta et al., 2022). Notably, these NLRs were downregulated post-inoculation, suggesting that suppression of NLR activity may contribute to susceptibility. The TNL (Soltu.DM.11G001840) contained two LRR-RLKs and four NLRs in its core-module (**Table 3**). Given that resistance to *Sss* is potentially polygenic, it will be valuable to examine the NLRs both individually and in combination. The TNL (Soltu.DM.11G001840) was also co-expressed with a RIN4 homolog (**Table 3**), a known ETI component (Toruño et al., 2019; Liu et al., 2009). In potato, RIN4 has been hypothesized to be involved in resistance to *Phytophthora infestans*. We identified RIN4 as associated with downregulated potential susceptibility factor, but the contribution of RIN4 to resistance to *Sss* remains unclear. In the previous transcriptomic study by Lekota et al. (2019), three NLRs associated with *Sss* infection were identified, although the lack of specific gene IDs prevented direct comparison with our findings. Nevertheless, our identification of additional candidate NLRs associated with *Sss* and PMTV infections further expands the potential for developing potato disease control strategies through enhanced plant immunity.

Four shared genes (Soltu.DM.01G038640.1, Soltu.DM.11G001490.1, Soltu.DM.11G003830.1, and Soltu.DM.09G009610.1 as shown with asterisk in **Table 3**) were identified among the core-modules of *Sss*-associated NLR Soltu.DM.11G001840.2 and Soltu.DM.11G002170.1, indicating the core-modules of these two candidates are connected. One of these connections, Soltu.DM.01G038640.1, is a potential PRR, and the connections among these candidates further support potential involvement in host response and highlight the importance of functional validation.

A TNL, Soltu.DM.06G004980.4, was identified in association with PMTV, suggesting that sensor-NLR-mediated recognition may play a role in PMTV defense. This NLR is linked to a known SA-related marker, PR10, in the co-expression network, providing evidence of involvement in SA-mediated resistance against PMTV and therefore a candidate for future validation.

### Potential involvement of glutathione S-transferase in host defense against *Sss* and PMTV

GSTs are multifunctional enzymes involved in detoxification, oxidative stress regulation, and responses to biotic and abiotic stresses (Islam et al., 2019; Dixon et al., 2002). GSTs are differentially regulated following interaction with bacterial, fungal, and viral pathogens (Gullner et al., 2018; Ahn et al., 2016; Ojaghian et al., 2015), potentially contributing to detoxification of pathogen-derived compounds and mitigation of oxidative stress (Gullner et al., 2018; Sappl et al., 2009). GST is also linked to SA signaling and may participate in SA perception or regulation (Gullner et al., 2018; Sappl et al., 2009; Tian et al., 2012). In this study, multiple GST genes were identified as potentially involved in defense against *Sss* and PMTV. Soltu.DM.06G000540 was downregulated in SH post-Sss inoculation, whereas Soltu.DM.01G029100 was upregulated in PR, but downregulated in PR following PMTV infection. Prior studies have speculated on GST involvement in *Sss* resistance (Balotf et al., 2025, 2022), but direct functional validation remains lacking. Our results strengthen this hypothesis and highlight GSTs as relevant targets for future studies.

### Potential involvement of Lipoxygenase in host defense against *Sss* and PMTV

Two 9S-lipoxygenases (9-LOXs), Soltu.DM.09G02418 (splice variants.2 and.3) associated with Sss infection and Soltu.DM.08G005440.4 associated with PMTV infection, were identified as a candidate gene in SH and RB using the DGE-GCN integrated pipeline. LOXs catalyzes the oxygenation of linoleic acid and linolenic acid, producing oxylipins such as 12-oxo-phytodienoic acid (OPDA), a key intermediate in JA biosynthesis pathway (Blée, 2002). Oxylipins are broadly recognized as important regulators of plant defense responses across diverse plant-pathogen systems (Blée, 2002). Indeed, LOX-mediated defense signaling has been implicated in responses to a wide range of pathogens, including *Meloidogyne javanica*, *Pseudomonas syringae* pv *tomato, Xanthomonas campestris* pv *vesicatoria, Colletotrichum coccodes, Hyaloperonospora arabidopsidis, Alternaria brassicicola* and *Phytophthora infestans* (Fitoussi et al., 2021; Vellosillo et al., 2007; Hwang and Hwang, 2010; Fauconnier et al., 2003; Kolomiets et al., 2000; Weber et al., 1999 Fauconnier). The involvement of LOX in response to *Sss* infection has also been reported by previous studies. Perla et al. (2014) reported that elevated LOX expression was negatively correlated with disease severity and positively associated with russet cultivars, suggesting that its potential as a marker for resistance breeding. Consistent with this, our prior work demonstrated increased accumulation of oxylipins OPDA and 9-hydroxy-10-oxo-12(Z),15(Z)-octadecadienoic acid (9,10-KODA), following *Sss* infection (Jayasinghe et al., 2025). Furthermore, an *OPR3* knockdown line (characterized by reduced JA but elevated OPDA levels) exhibited significantly reduced root galling symptoms. This finding suggests that OPDA-dependent signaling, rather than JA itself, may play a critical role in defense under certain conditions (Jayasinghe et al., 2025). Collectively, these observations suggest that LOX-derived oxylipins contribute to resistance against *Sss* and potentially also PMTV.

In addition to their role in lipid-derived signaling, LOXs modulate ROS and contribute to plant defense through activation of defense-related programmed cell death and reindorsement of structural barriers, e.g., suberin deposition (Perla et al., 2014). In parallel, GSTs likely help maintain cellular redox homeostasis, limiting oxidative damage during defense activation. Together, LOX- and GST-associated processes may shape the cellular redox environment that supports multiple defense outputs, thereby restricting pathogen ingress. This interpretation is supported by our data, which show functional enrichment of DEGs associated with ROS-, cell wall-, and cell death-related pathways (**Supplementary Figures 16**-**21**, **S26**, **S27**).

Within this framework, LOX emerges as a central integrator of chemical and structural defense mechanisms. By linking oxylipin signaling, redox regulation with GSTs, and physical barrier formation (i.e., suberin), LOX-mediated pathways likely contribute to a multifaceted immune strategy that enhances plant resilience against *Sss* and PMTV complexes.

## Conclusions and future directions

This study aimed to identify gene candidates contributing to resistance against *Sss* and PMTV, thereby providing a molecular toolkit for resistance breeding. We identified 80 high-confidence candidate genes (52 associated with *Sss* infection and 28 with PMTV infection). These findings substantially advance our understanding of host responses and provide the scientific community with potential avenues to control two pathogens that are becoming an increasing threat to global potato production. Functional validation of these genes is essential before their development for breeding or genetic engineering. Future work will focus on molecular genetic validation (e.g., using gene overexpression, RNA interference, and CRISPR-mediated knockout) to determine causal roles in disease resistance and susceptibility. Validated genes will be robust targets for introgression into elite potato cultivars through breeding or biotechnology-based approaches. Additionally, identified genes may serve as molecular markers to accelerate selection in resistance-breeding programs. Developing potato cultivars that combine agronomic desirability with durable resistance will be critical for sustainable management of powdery scab and PMTV, ultimately helping to mitigate their economic impact on global potato production.

## Data Availability

The transcriptome datasets presented in this study can be found in the Mendeley Data repository at Jayasinghe and Tanaka (2026) https://doi.org/10.17632/tv6mjd9c95.1 and also in NCBI BioProject under the accession number PRJNA1108886.
