# Supplementary material for: Dynamic transcriptomic responses reveal candidate defense genes against Spongospora subterranea f. sp. subterranea and Potato mop-top virus infection
Source: Front Plant Sci. 2026 Jun 11;17:1799568. doi: 10.3389/fpls.2026.1799568 (PMC13294368; doi:10.3389/fpls.2026.1799568)
Supplement: Supplementary file 3 [file Presentation1.pdf]

Supplementary Materials for

Dynamic transcriptomic responses reveal candidate defense genes against *Spongospora subterranea* f. sp. *subterranea* and Potato mop-top virus infection

Samodya K. Jayasinghe,[1] Natalia Moroz,[1] Stephen P. Ficklin,[2] Kiwamu Tanaka[1]*

Department of Plant Pathology, Washington State University, Pullman, Washington 99164;

Department of Horticulture, Washington State University, Pullman, Washington 99164;

*For correspondence: kiwamu.tanaka@wsu.edu

The PDF file includes:

Supplementary Figures: S1 to S31

Supplementary Tables: S1 to S2

Other Supplementary Material for this manuscript includes the following:

Supplemantary Data 1

Supplemantary Data 2

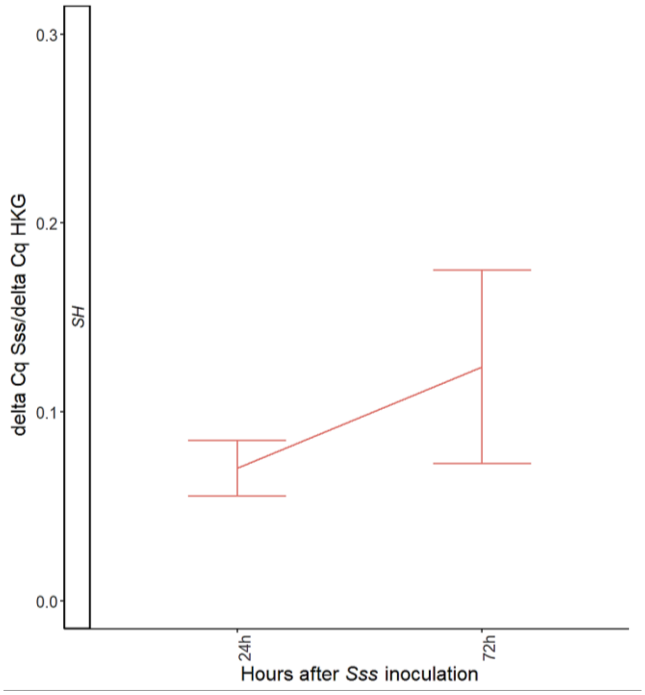

Supplementary Figure S1. Detection of *Sss* in hairy roots of potato cultivar Shepody within 72 hours post-inoculation, representing early stages of infection. *Sss* ITS2 DNA was quantified by qPCR in root tissues and normalized to geometric mean of two potato housekeeping genes (HKGs): *Ef1-alpha* and *Ubiquitin.*

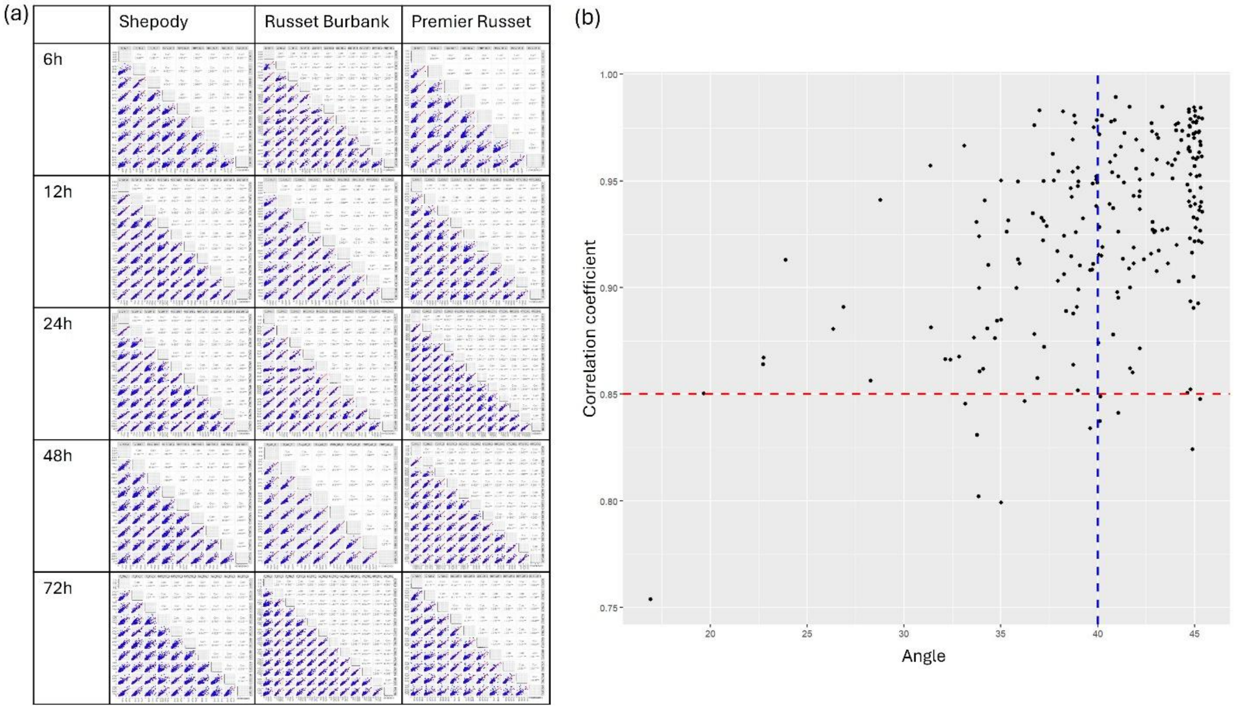

Supplementary Figure S2. Data processing for outlier removal. (a) Scatterplots showing the linear relationships and correlation coefficients among all samples for each cultivar at each time point. (b) Scatterplot of correlation coefficients versus the regression line angle (relative to the x-axis) for all replicates within each cultivar × treatment × time point combination. Samples with correlation coefficients > 0.85 with all other replicates in their group and/or regression line angles > 40° were retained for downstream analysis.

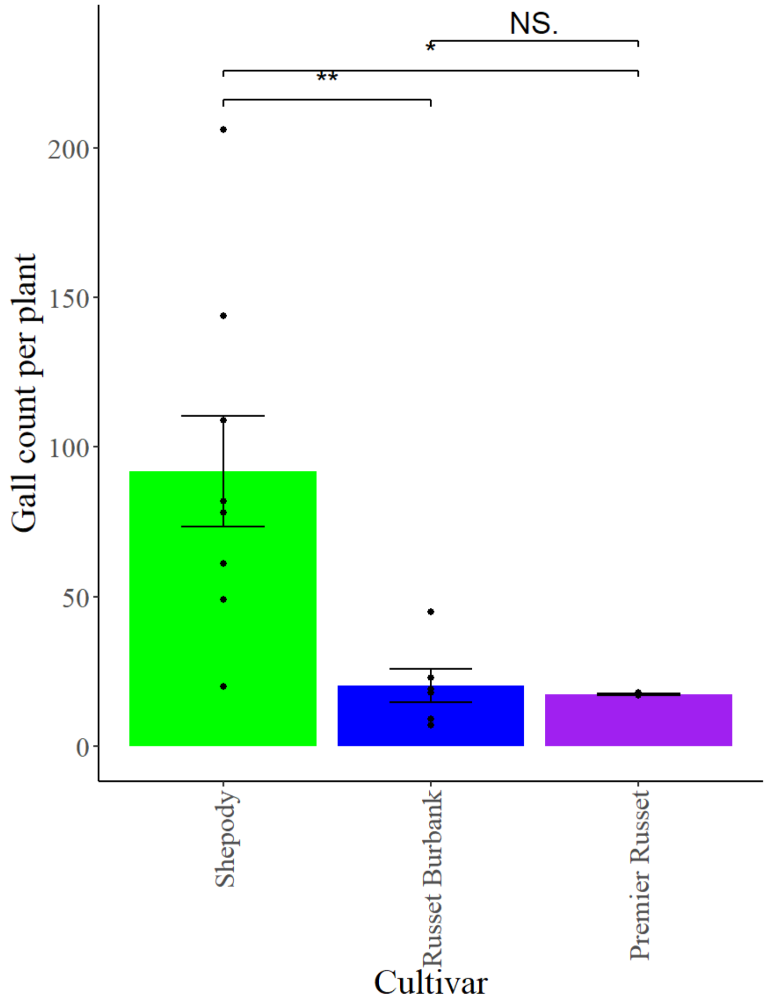

Supplementary Figure S3. Quantification of root gall formation as a measure of disease severity caused by *Sss* infection in three potato cultivars used in this study. Plants were grown in a controlled growth chamber under 18 °C/16 °C day/night temperatures with a 12h/12h day/night photoperiod. Root galls were counted at 90 days post-inoculation.

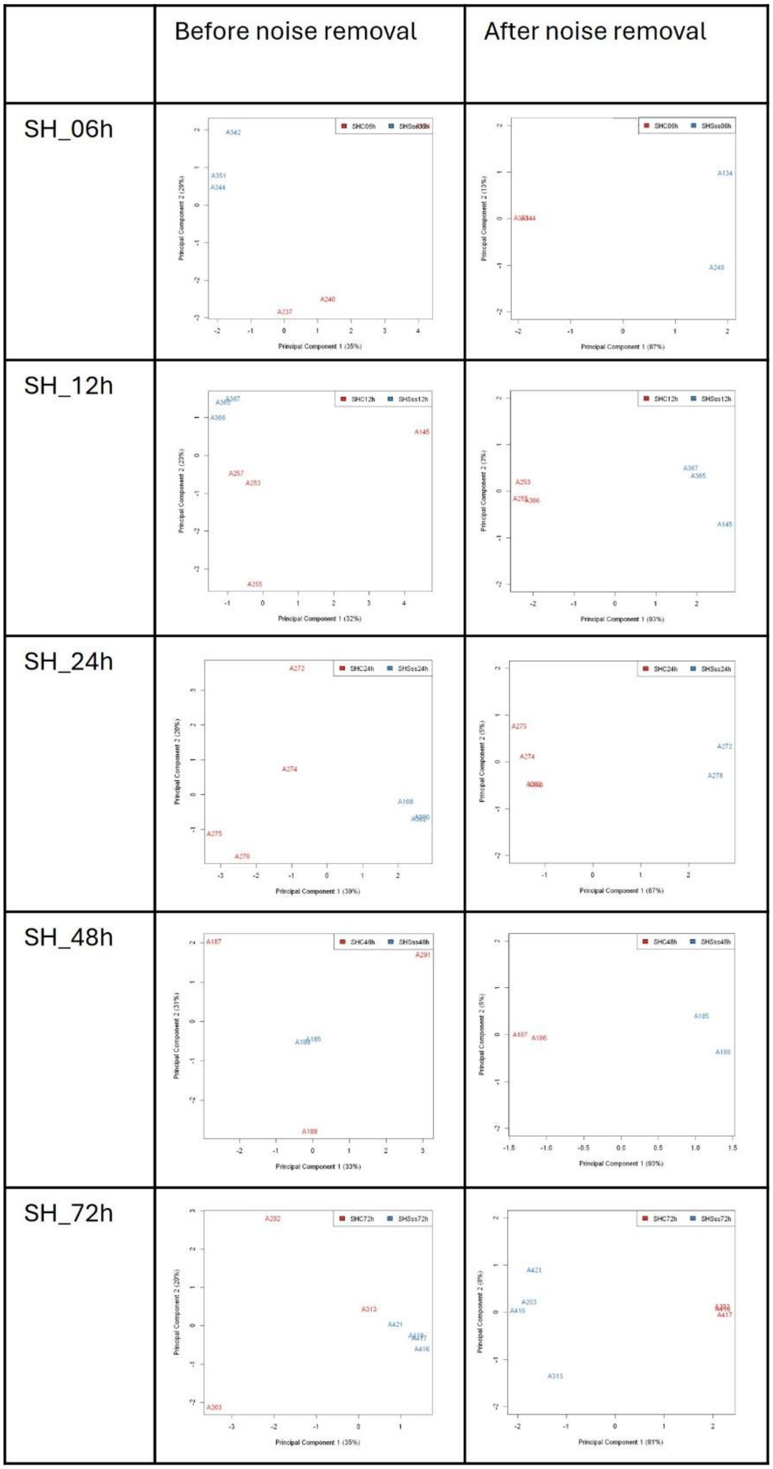

Supplementary Figure S4. PCA plots of Shepody (SH) samples before and after outlier removal and noise correction using noisyR. Red points represent mock treated samples, and blue points represent samples inoculated with non-viruleferous *Sss*.

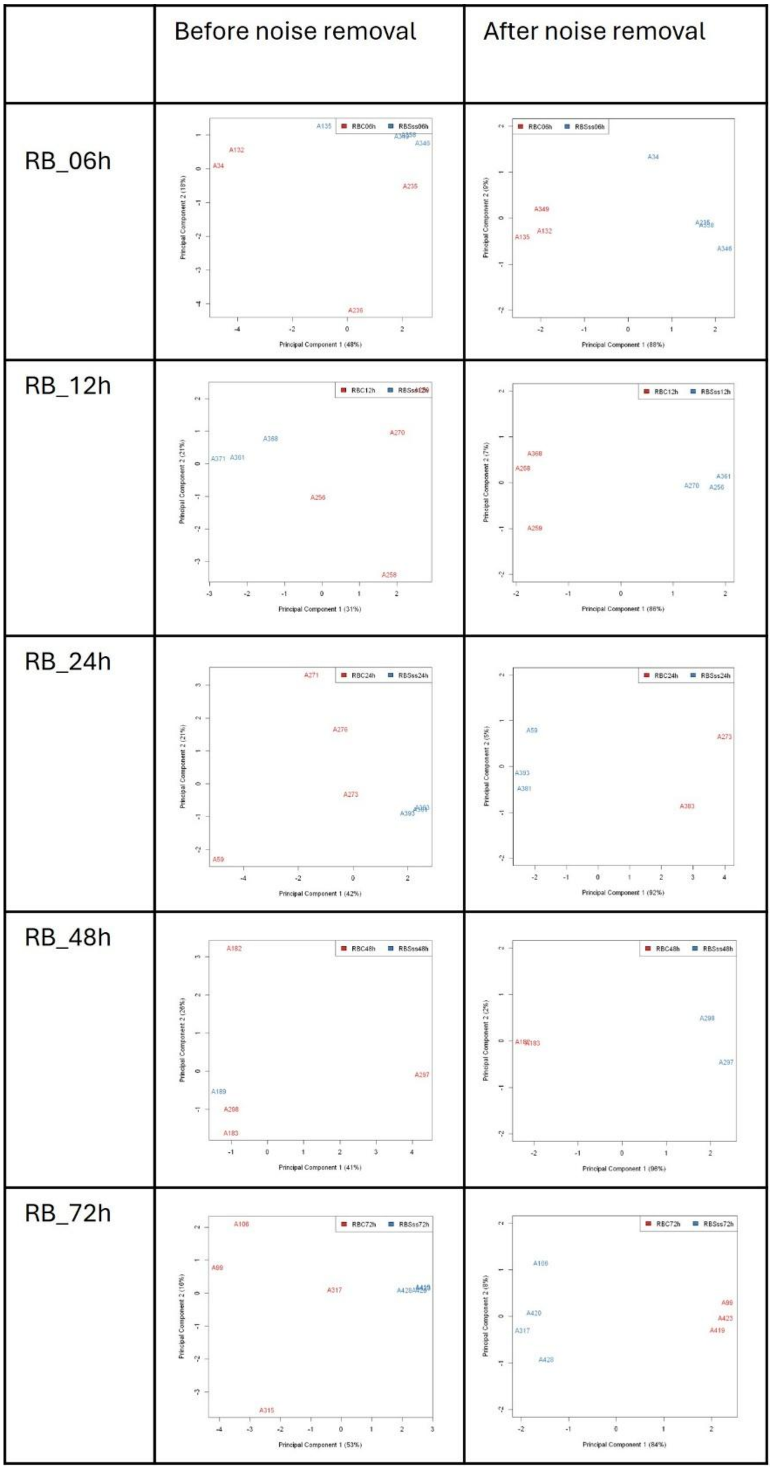

Supplementary Figure S5. PCA plots of russet Burbank (RB) samples before and after outlier removal and noise correction using noisyR. Red points represent mock treated samples, and blue points represent samples inoculated with non-viruleferous *Sss*.

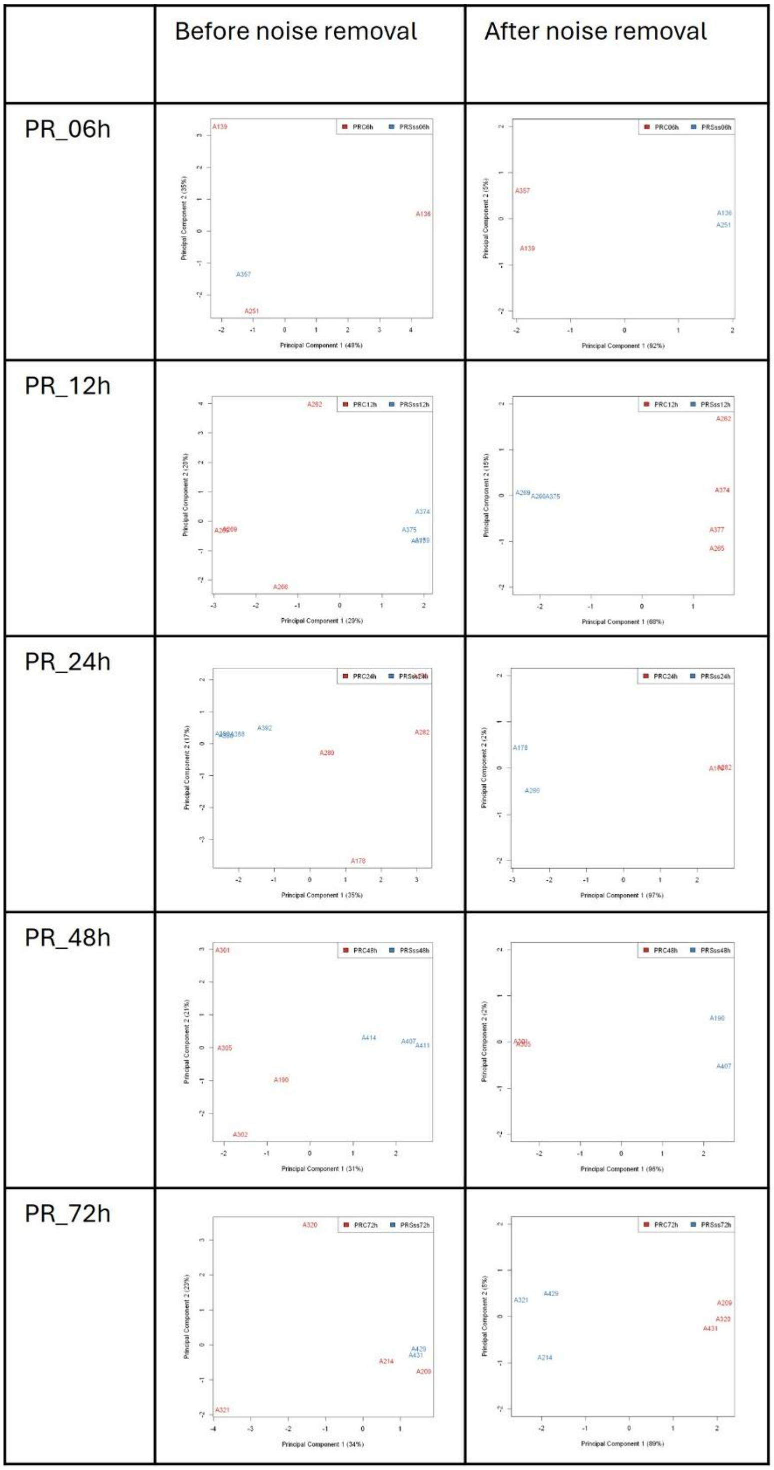

Supplementary Figure S6. PCA plots of Premier russet (PR) samples before and after outlier removal and noise correction using noisyR. Red points represent mock treated samples, and blue points represent samples inoculated with non-viruleferous *Sss*.

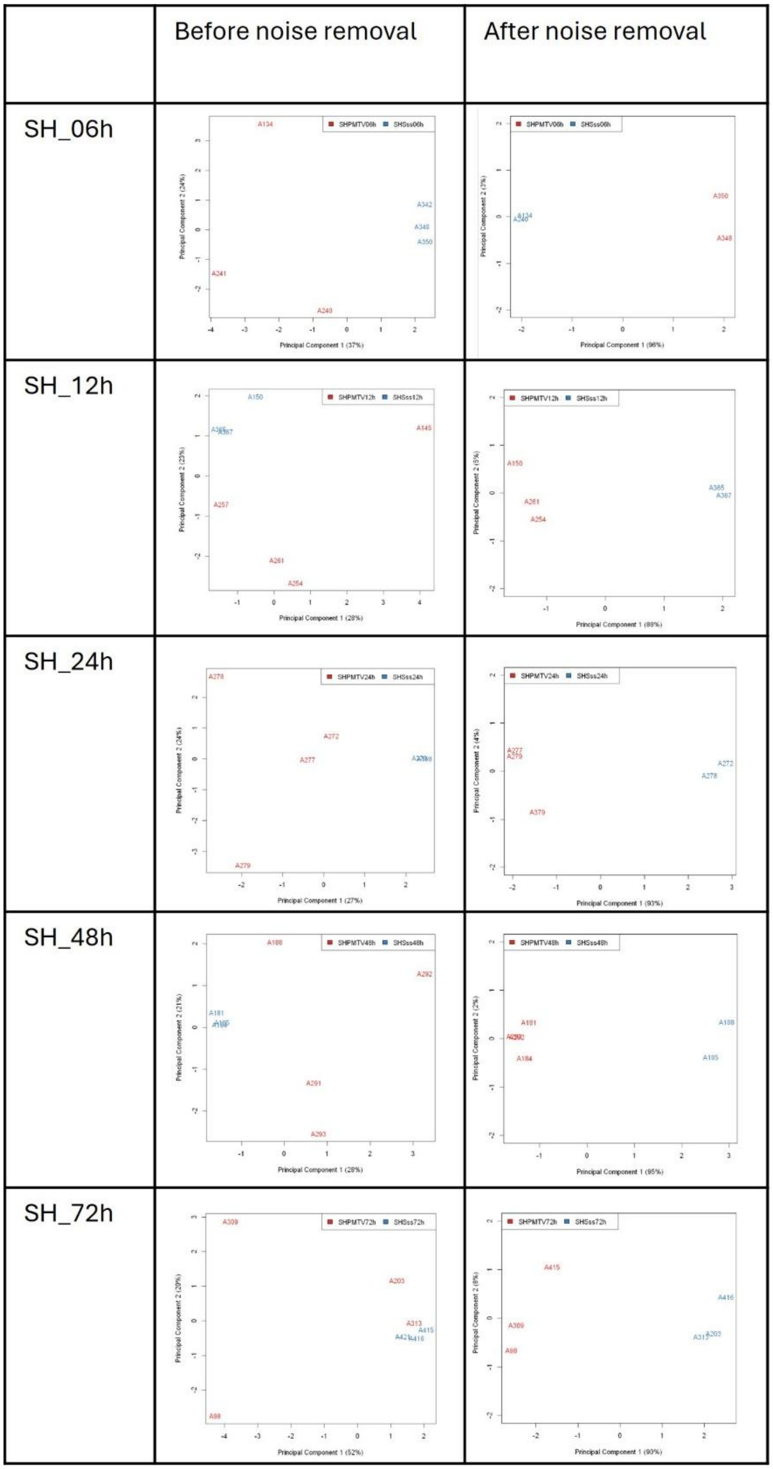

Supplementary Figure S7. PCA plots of Shepody (SH) samples before and after outlier removal and noise correction using noisyR. Red points represent samples inoculated with PMTV-carrying *Sss*, and blue points represent samples inoculated with non-viruleferous *Sss*.

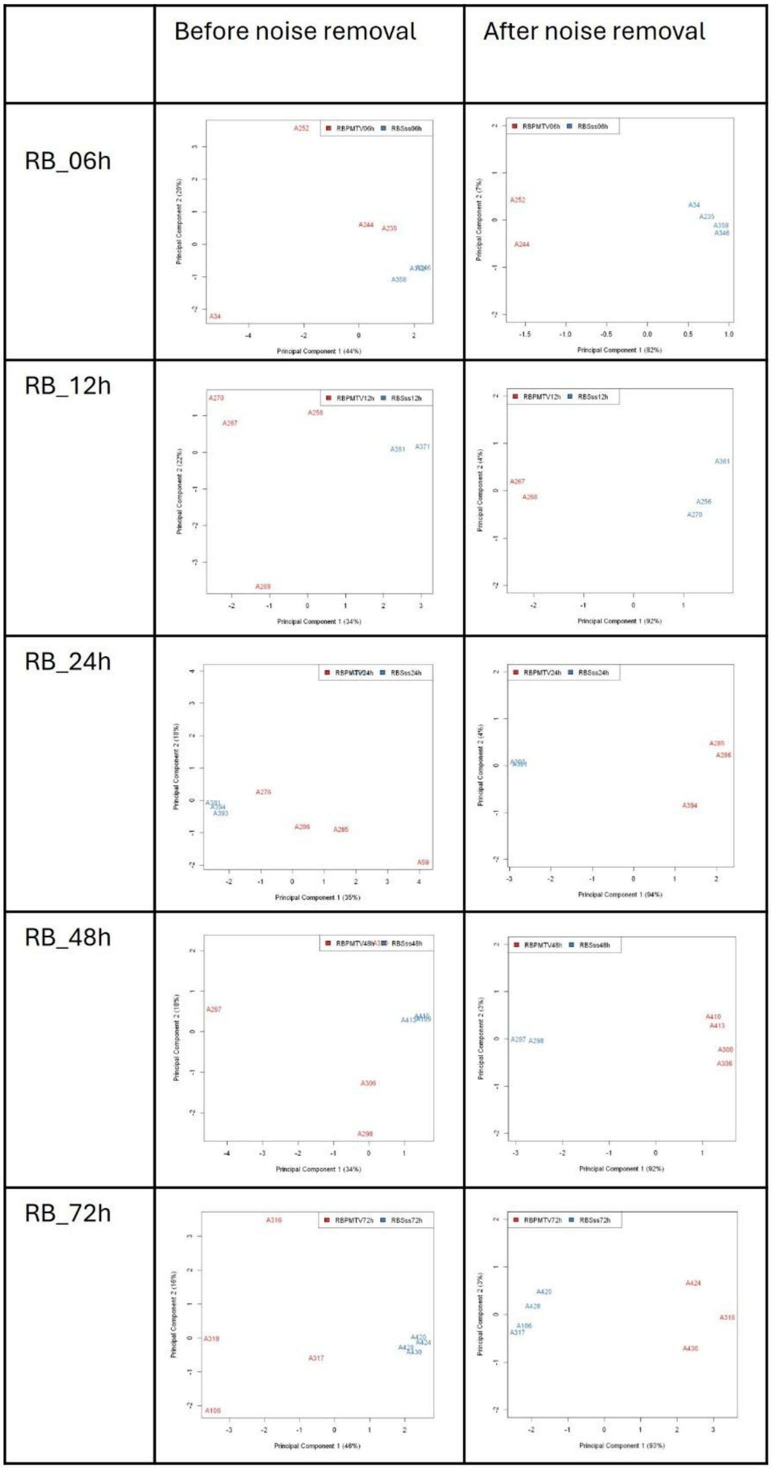

Supplementary Figure S8. PCA plots of Russet Burbank (RB) samples before and after outlier removal and noise correction using noisyR. Red points represent samples inoculated with PMTV-carrying *Sss*, and blue points represent samples inoculated with non-viruleferous *Sss*.

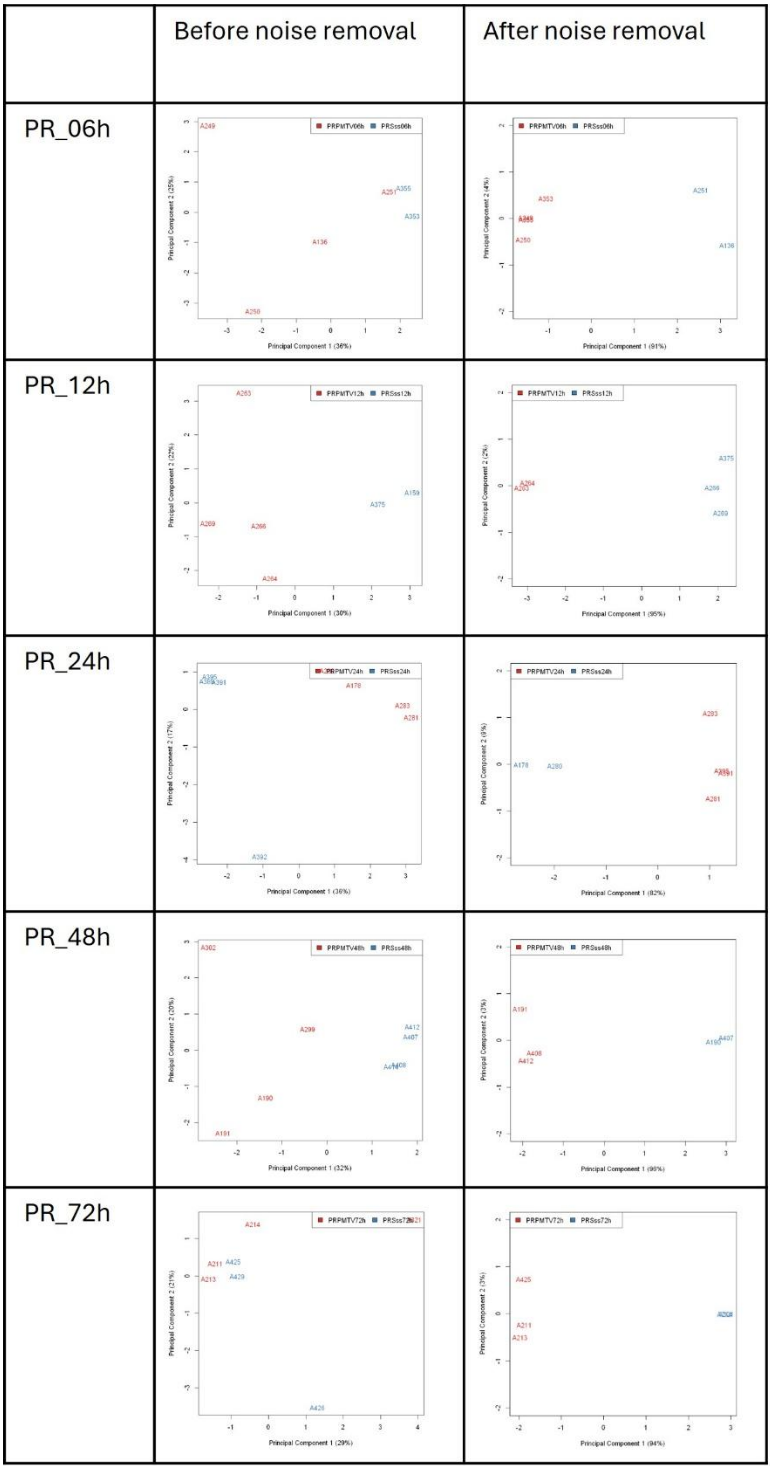

Supplementary Figure S9. PCA plots of Premier Russet (PR) samples before and after outlier removal and noise correction using noisyR. Red points represent samples inoculated with PMTV-carrying *Sss*, and blue points represent samples inoculated with non-viruleferous *Sss*.

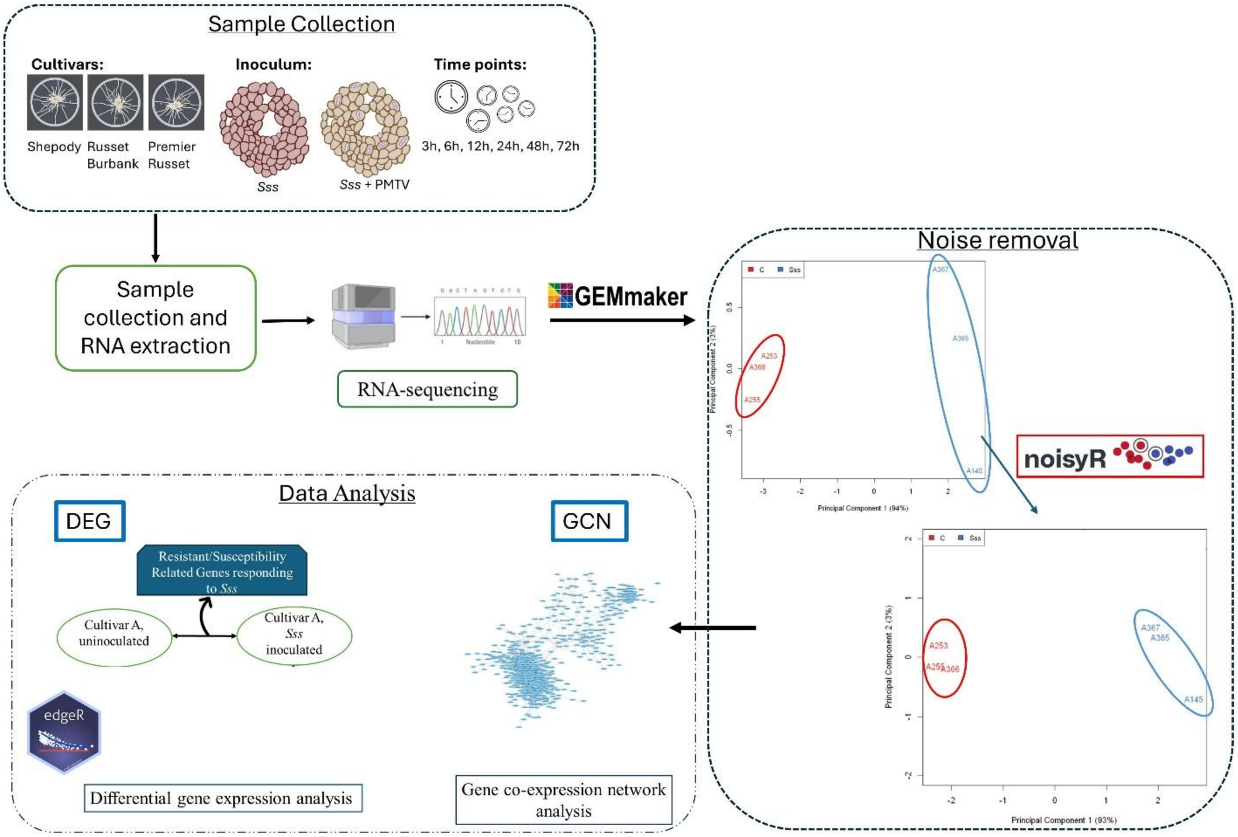

Supplementary Figure S10. Overview of the transcriptomics workflow, from sample collection to data analysis. The GEMmaker pipeline was used for sample quality control, adaptor trimming, annotation to the reference genome, expression quantification, and Gene Expression Matrix (GEM) construction, followed by outlier removal (Figure S2) and noise correction (Figures S3–S8). The cartoons of *S. subterranea* sporosori depict non-viruliferous *Sss* and *Sss* carrying PMTV, which were used for inoculation.

(a)

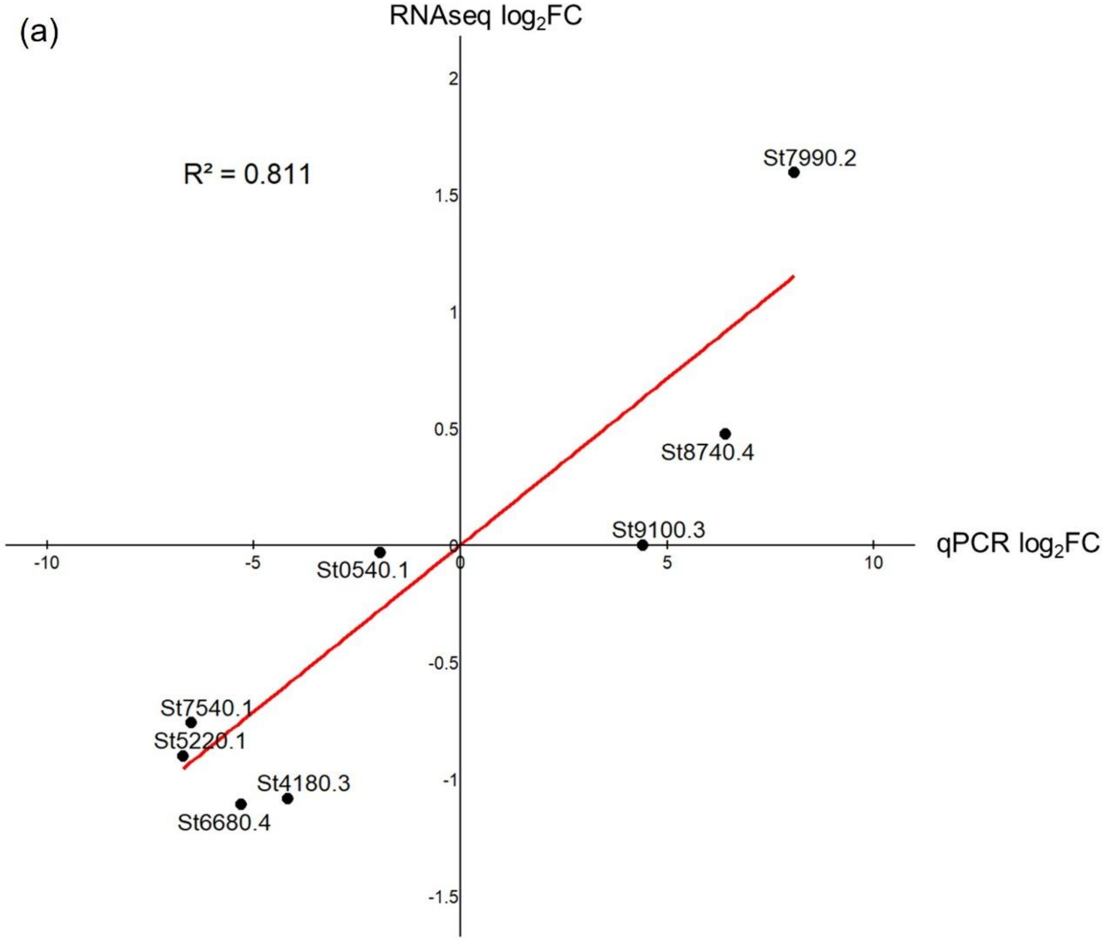

(b)

Cultivar	Time point	Regulation	Gene abbreviation	Gene	RNAseq $\log_2$FC	RTqPCR $\log_2$FC
PR	06h	Up	St9100.3	Soltu.DM.01G029100.3	4.423	0
PR	48h	Up	St8740.4	Soltu.DM.01G048740.4	6.405	0.473
PR	48h	Up	St7990.2	Soltu.DM.02G017990.2	8.083	0.006
SH	06h	Down	St0540.1	Soltu.DM.06G000540.1	-1.977	-0.033
SH	12h	Down	St5220.1	Soltu.DM.01G035220.1	-6.719	-0.902
SH	12h	Down	St6680.4	Soltu.DM.06G016680.4	-5.312	-0.005
SH	24h	Down	St7540.1	Soltu.DM.01G037540.1	-6.504	-0.761
SH	24h	Down	St4180.3	Soltu.DM.09G024180.3	-4.175	-1.085

Supplementary Figure S11. Validation of selected candidate genes associated with *Sss* infection using RT-qPCR. (a) Correlation between RT-qPCR data and RNA-seq data for eight selected candidate candidates. Values are presented as $\log_2$FC values. (b) Gene IDs of the validated candidates along with their corresponding $\log_2$FC values.

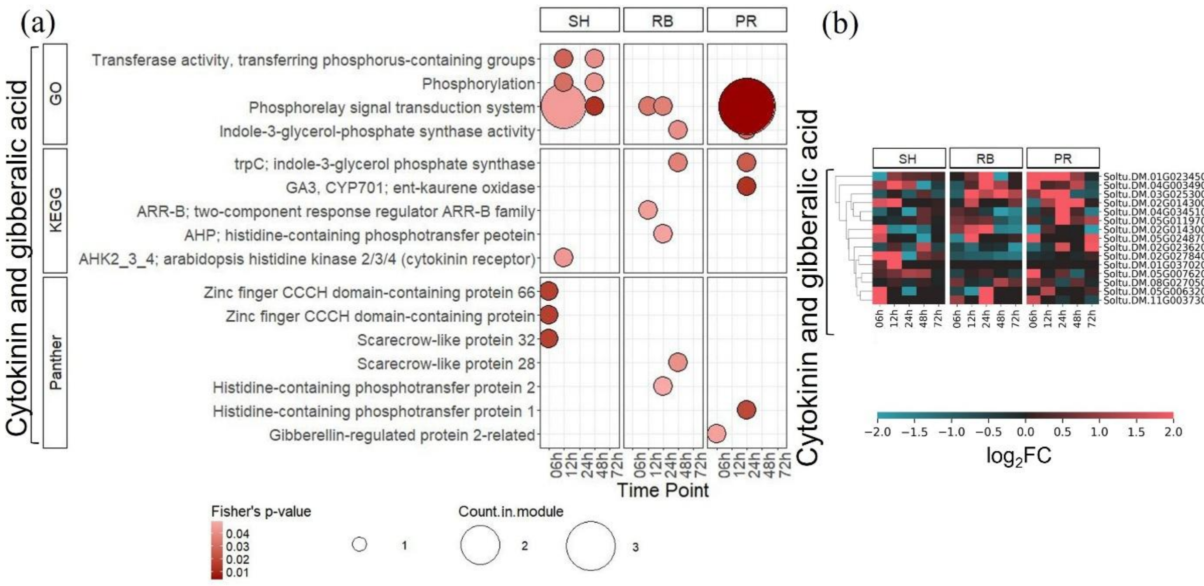

Supplementary Figure S12. Functional enrichments of upregulated DEGs associated with *Sss* infection in cytokinin- and gibberellic acid-related pathways. (a) Bubble plots showing significantly enriched, upregulated functional terms from GO, KEGG, and PANTHER ontology analyses. (b) Heatmaps showing expression patterns of genes with each enriched functional category, identified as responsive to *Sss* infection through DGE analysis. The x-axis indicates the the time points of sample collection post-pathogen inoculation across different potato cultivars. SH: Shepody, RB: Russet Burbank, PR: Premier Russet.

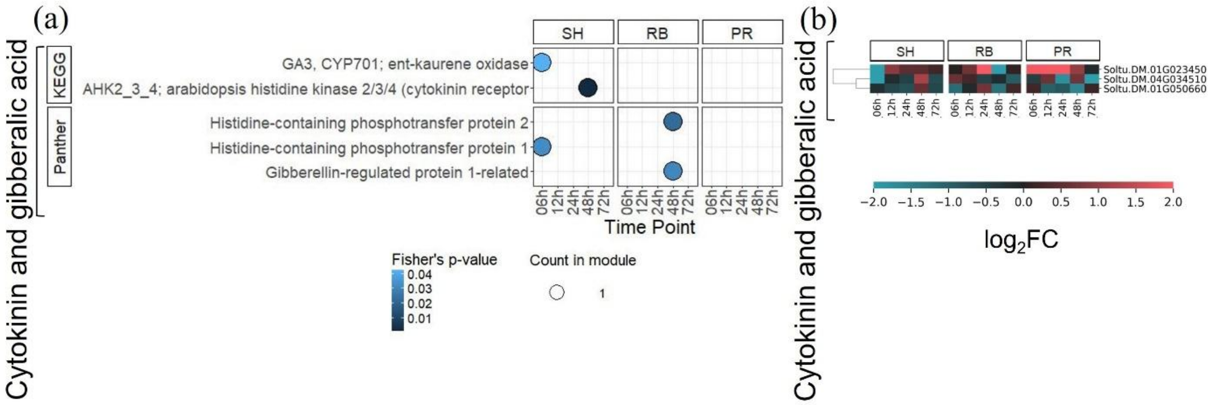

Supplementary Figure S13. Functional enrichments of downregulated DEGs associated with *Sss* infection in cytokinin- and gibberellic acid-related pathways. (a) Bubble plots showing significantly enriched, upregulated functional terms from GO, KEGG, and PANTHER ontology analyses. (b) Heatmaps showing expression patterns of genes with each enriched functional category, identified as responsive to *Sss* infection through DGE analysis. The x-axis indicates the the time points of sample collection post-pathogen inoculation across different potato cultivars. SH: Shepody, RB: Russet Burbank, PR: Premier Russet.

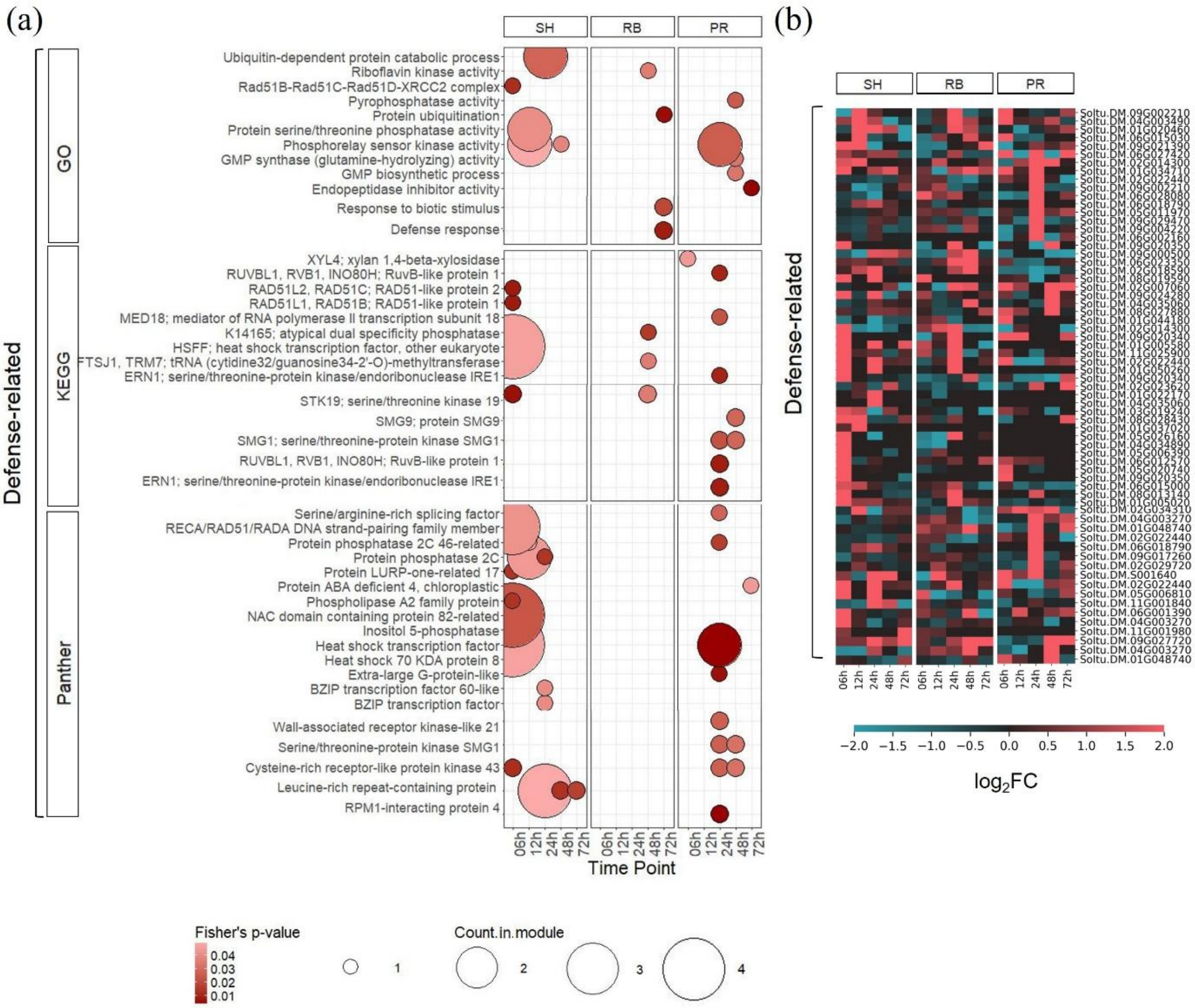

Supplementary Figure S14. Functional enrichments of upregulated DEGs associated with *Sss* infection in defense-related pathways. (a) Bubble plots showing significantly enriched, upregulated functional terms from GO, KEGG, and PANTHER ontology analyses. (b) Heatmaps showing expression patterns of genes with each enriched functional category, identified as responsive to *Sss* infection through DGE analysis. The x-axis indicates the the time points of sample collection post-pathogen inoculation across different potato cultivars. SH: Shepody, RB: Russet Burbank, PR: Premier Russet.

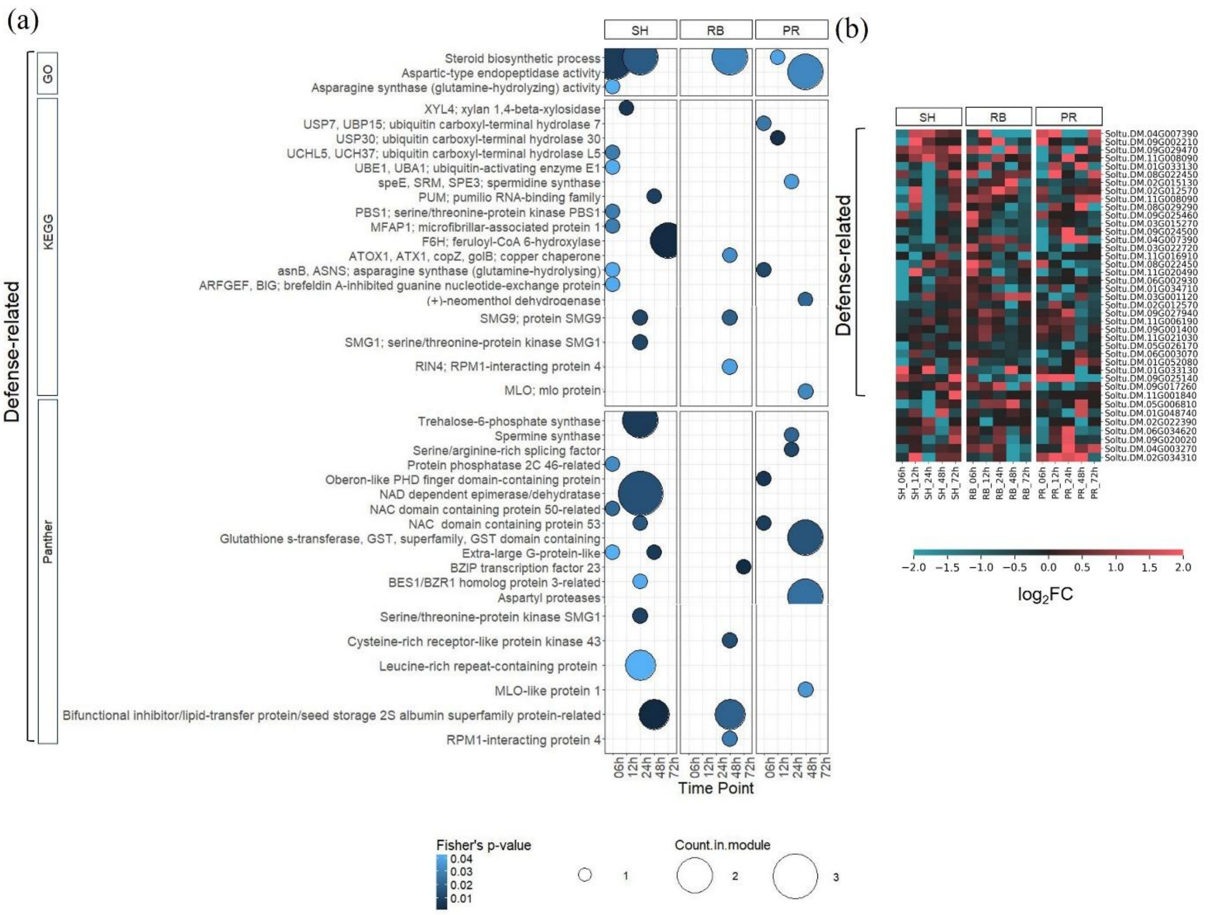

Supplementary Figure S15. Functional enrichments of downregulated DEGs associated with *Sss* infection in defense-related pathways. (a) Bubble plots showing significantly enriched, upregulated functional terms from GO, KEGG, and PANTHER ontology analyses. (b) Heatmaps showing expression patterns of genes with each enriched functional category, identified as responsive to *Sss* infection through DGE analysis. The x-axis indicates the the time points of sample collection post-pathogen inoculation across different potato cultivars. SH: Shepody, RB: Russet Burbank, PR: Premier Russet.

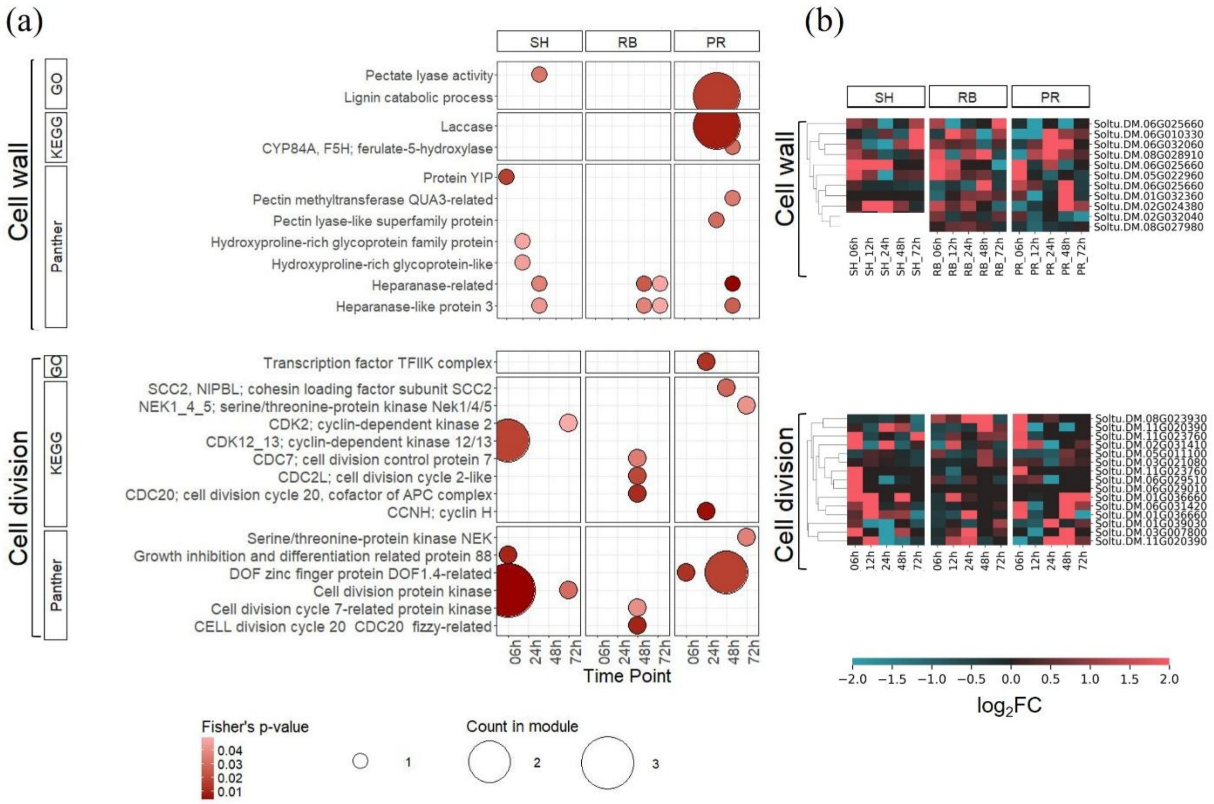

Supplementary Figure S16. Functional enrichments of upregulated DEGs associated with *Sss* infection in cell wall- and cell division-related pathways. (a) Bubble plots showing significantly enriched, upregulated functional terms from GO, KEGG, and PANTHER ontology analyses. (b) Heatmaps showing expression patterns of genes with each enriched functional category, identified as responsive to *Sss* infection through DGE analysis. The x-axis indicates the time points of sample collection post-pathogen inoculation across different potato cultivars. SH: Shepody, RB: Russet Burbank, PR: Premier Russet.

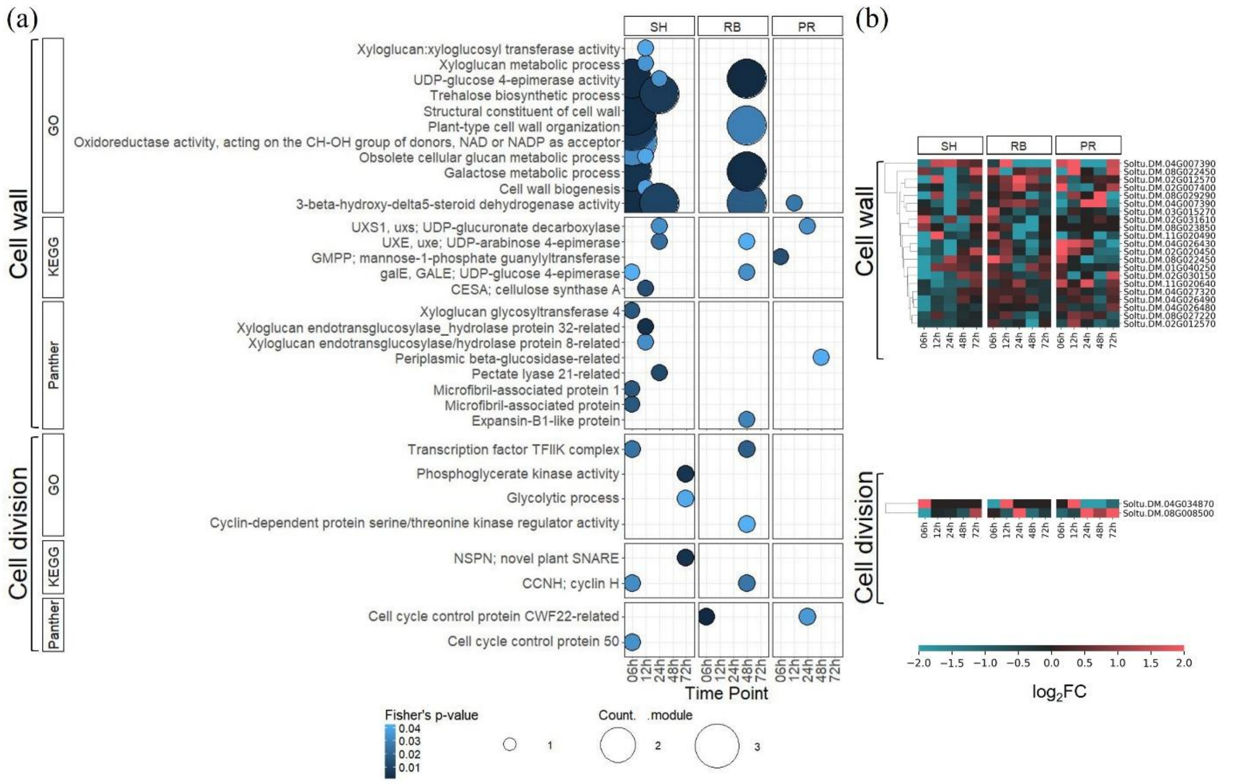

Supplementary Figure S17. Functional enrichments of downregulated DEGs associated with *Sss* infection in cell wall- and cell division-related pathways. (a) Bubble plots showing significantly enriched, upregulated functional terms from GO, KEGG, and PANTHER ontology analyses. (b) Heatmaps showing expression patterns of genes with each enriched functional category, identified as responsive to *Sss* infection through DGE analysis. The x-axis indicates the the time points of sample collection post-pathogen inoculation across different potato cultivars. SH: Shepody, RB: Russet Burbank, PR: Premier Russet.

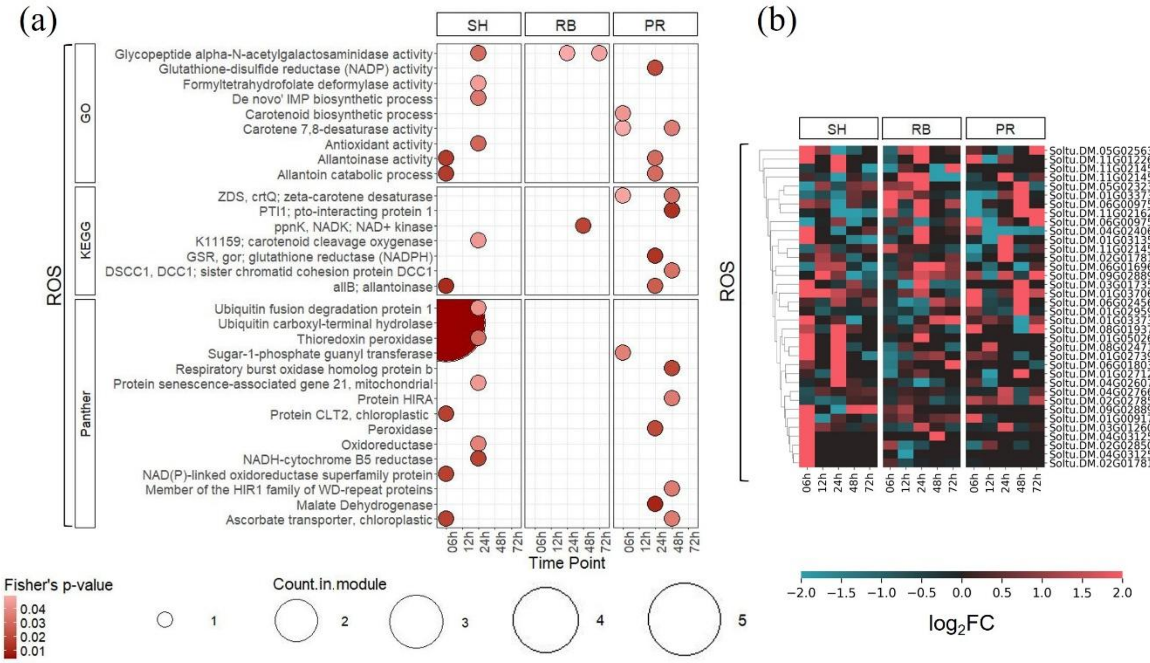

Supplementary Figure S18. Functional enrichments of upregulated DEGs associated with *Sss* infection in ROS-related pathways. (a) Bubble plots showing significantly enriched, upregulated functional terms from GO, KEGG, and PANTHER ontology analyses. (b) Heatmaps showing expression patterns of genes with each enriched functional category, identified as responsive to *Sss* infection through DGE analysis. The x-axis indicates the the time points of sample collection post-pathogen inoculation across different potato cultivars. SH: Shepody, RB: Russet Burbank, PR: Premier Russet.

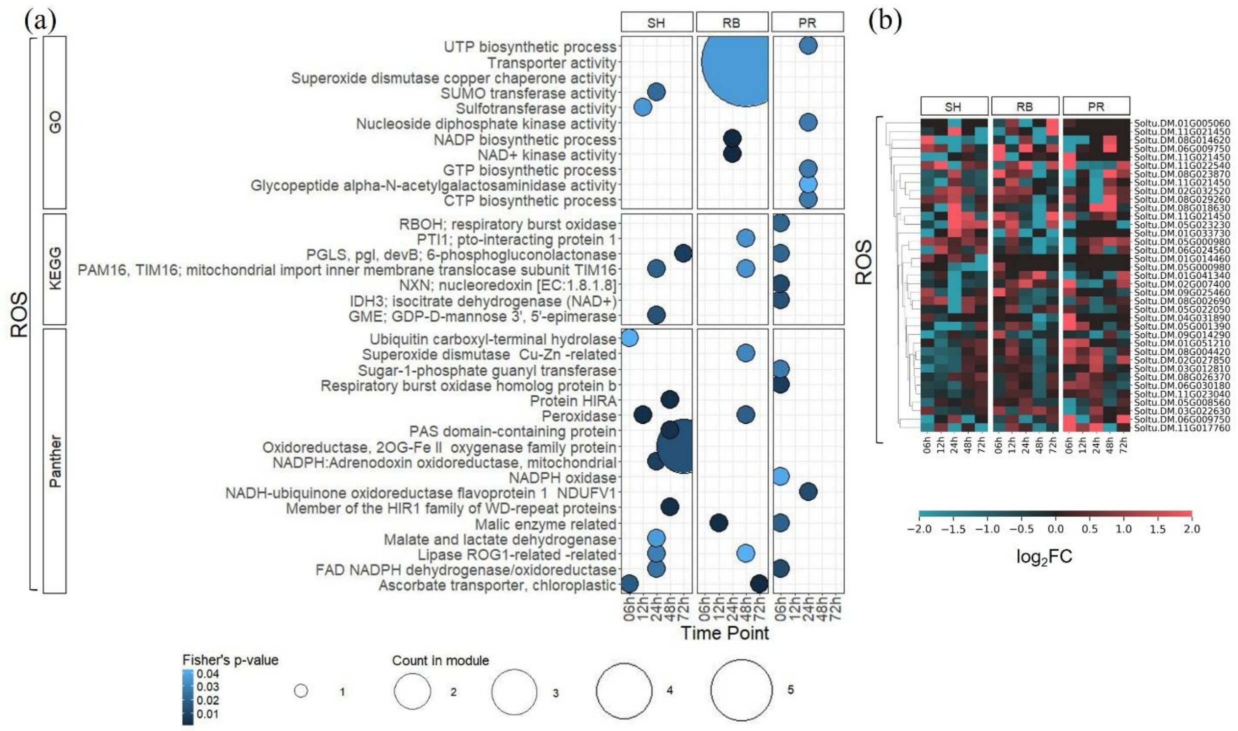

Supplementary Figure S19. Functional enrichments of downregulated DEGs associated with *Sss* infection in ROS-related pathways. (a) Bubble plots showing significantly enriched, upregulated functional terms from GO, KEGG, and PANTHER ontology analyses. (b) Heatmaps showing expression patterns of genes with each enriched functional category, identified as responsive to *Sss* infection through DGE analysis. The x-axis indicates the the time points of sample collection post-pathogen inoculation across different potato cultivars. SH: Shepody, RB: Russet Burbank, PR: Premier Russet.

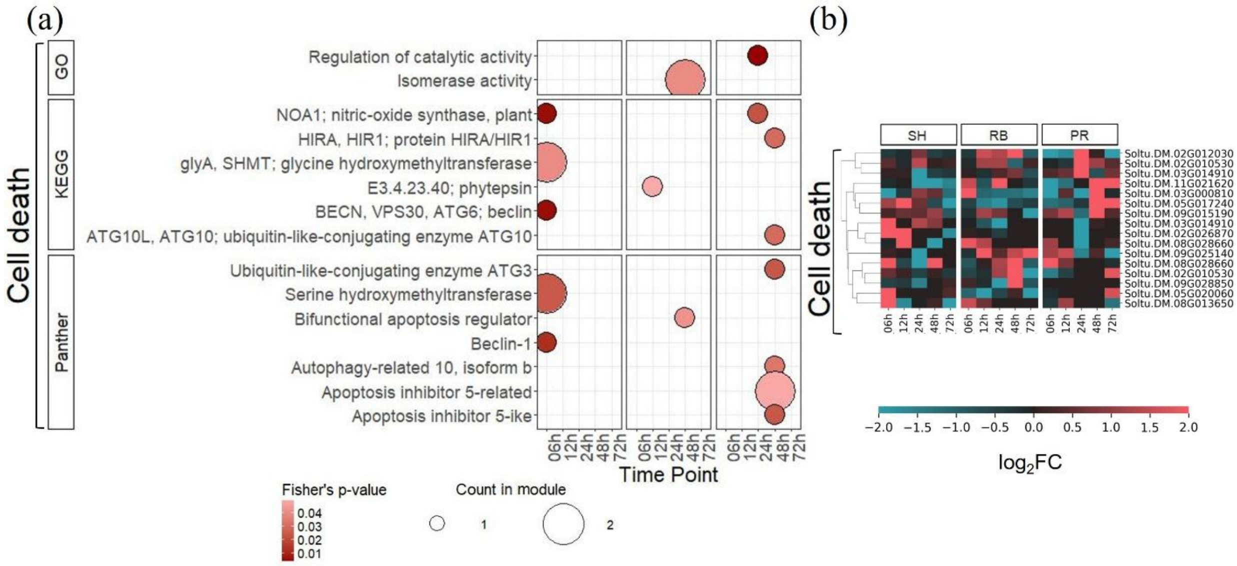

Supplementary Figure S20. Functional enrichments of upregulated DEGs associated with *Sss* infection in cell death-related pathways. (a) Bubble plots showing significantly enriched, upregulated functional terms from GO, KEGG, and PANTHER ontology analyses. (b) Heatmaps showing expression patterns of genes with each enriched functional category, identified as responsive to *Sss* infection through DGE analysis. The x-axis indicates the the time points of sample collection post-pathogen inoculation across different potato cultivars. SH: Shepody, RB: Russet Burbank, PR: Premier Russet.

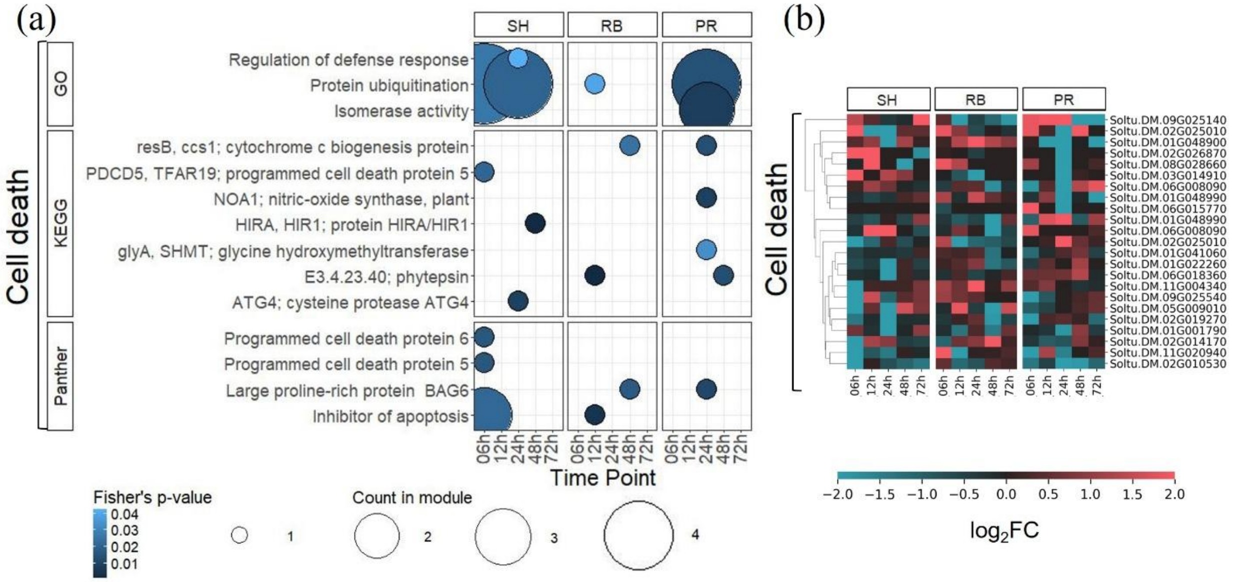

Supplementary Figure S21. Functional enrichments of downregulated DEGs associated with *Sss* infection in cell death-related pathways. (a) Bubble plots showing significantly enriched, upregulated functional terms from GO, KEGG, and PANTHER ontology analyses. (b) Heatmaps showing expression patterns of genes with each enriched functional category, identified as responsive to *Sss* infection through DGE analysis. The x-axis indicates the the time points of sample collection post-pathogen inoculation across different potato cultivars. SH: Shepody, RB: Russet Burbank, PR: Premier Russet.

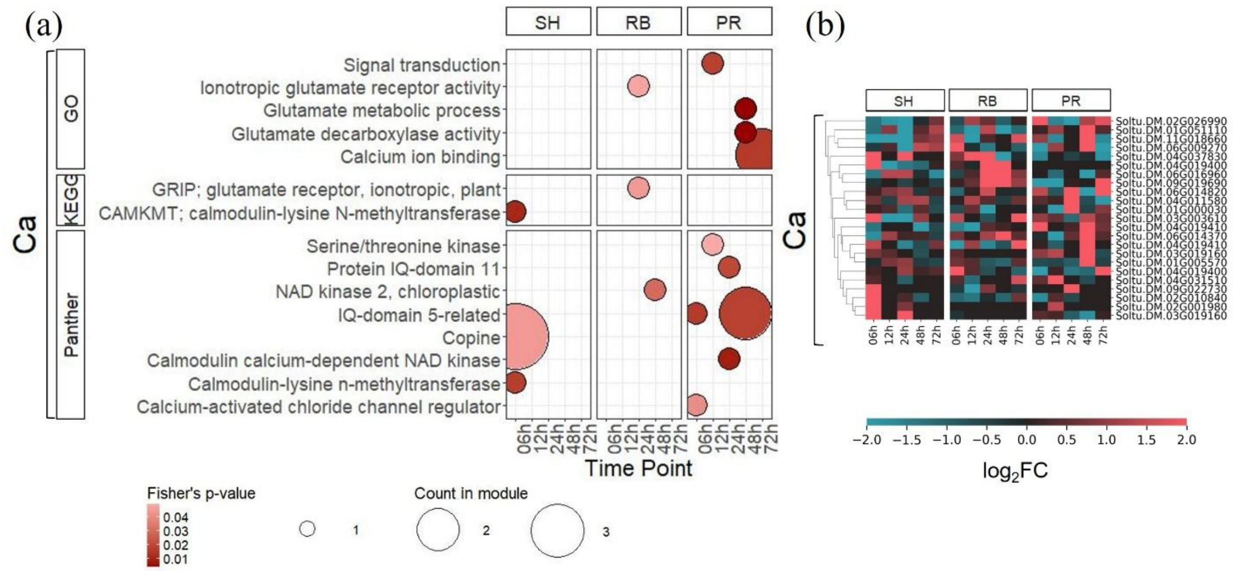

Supplementary Figure S22. Functional enrichments of upregulated DEGs associated with *Sss* infection in calcium (Ca)-related pathways. (a) Bubble plots showing significantly enriched, upregulated functional terms from GO, KEGG, and PANTHER ontology analyses. (b) Heatmaps showing expression patterns of genes with each enriched functional category, identified as responsive to *Sss* infection through DGE analysis. The x-axis indicates the the time points of sample collection post-pathogen inoculation across different potato cultivars. SH: Shepody, RB: Russet Burbank, PR: Premier Russet.

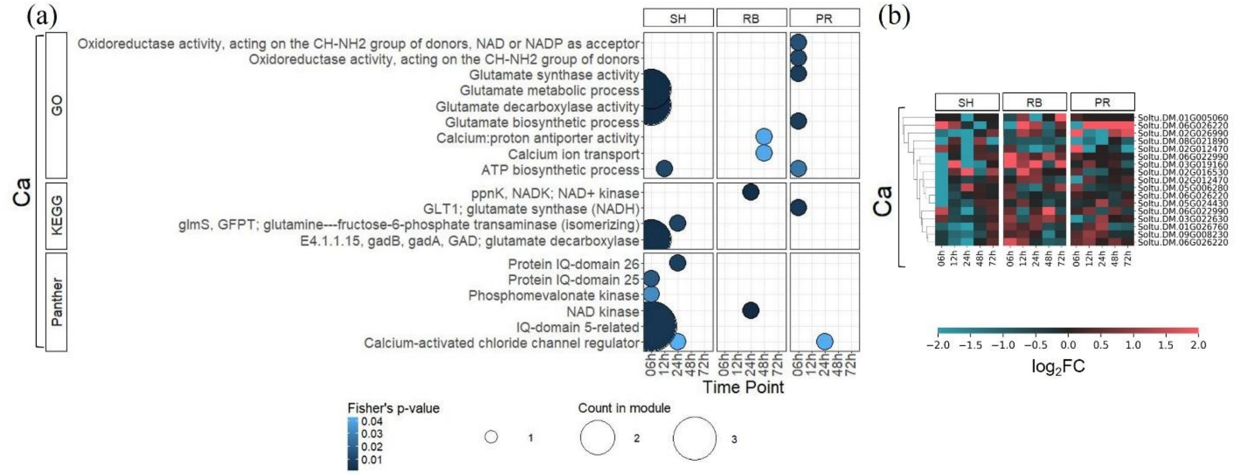

Supplementary Figure S23. Functional enrichments of downregulated DEGs associated with *Sss* infection in calcium (Ca)-related pathways. (a) Bubble plots showing significantly enriched, upregulated functional terms from GO, KEGG, and PANTHER ontology analyses. (b) Heatmaps showing expression patterns of genes with each enriched functional category, identified as responsive to *Sss* infection through DGE analysis. The x-axis indicates the the time points of sample collection post-pathogen inoculation across different potato cultivars. SH: Shepody, RB: Russet Burbank, PR: Premier Russet.

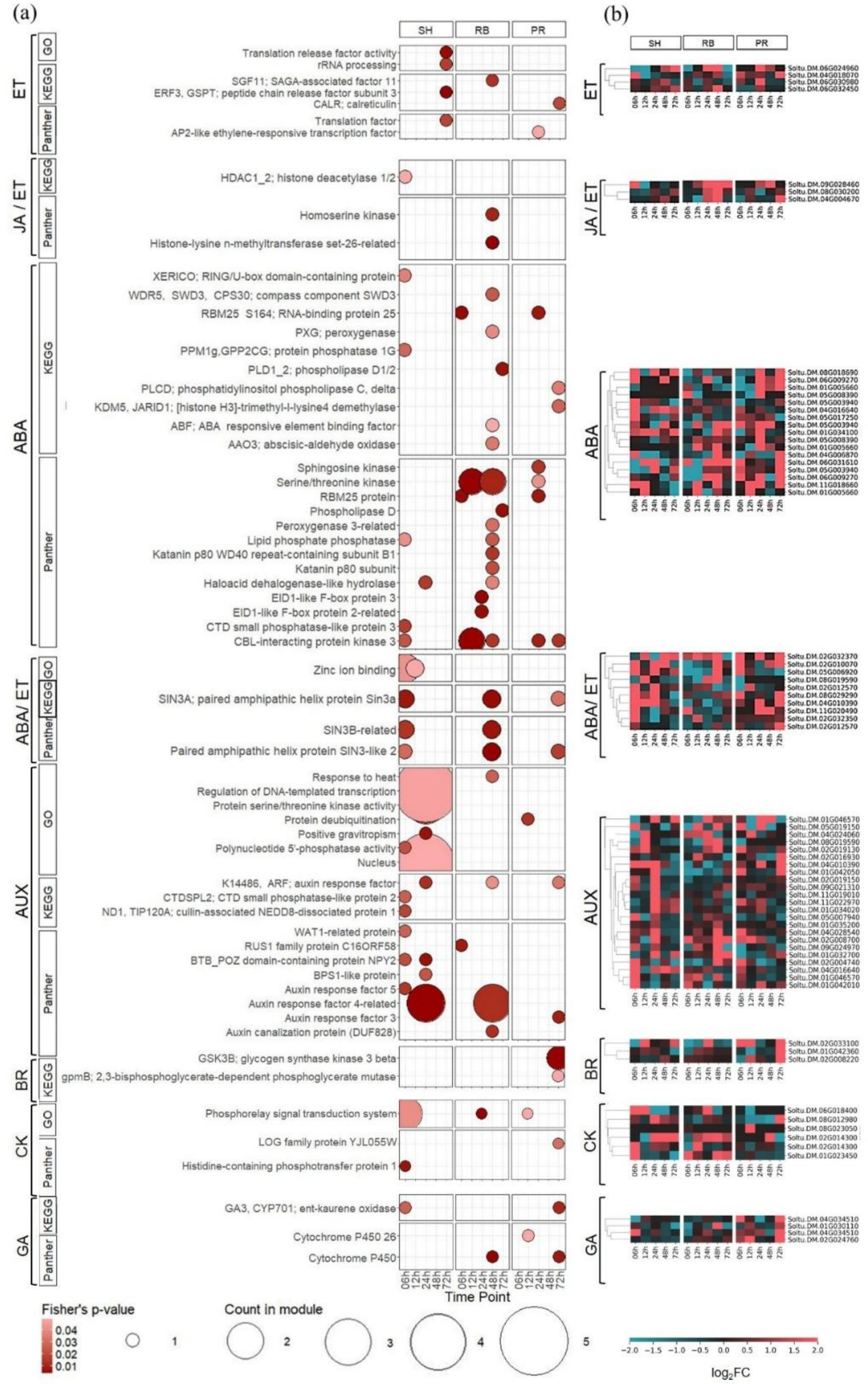

Supplementary Figure S24. Functional enrichments of upregulated DEGs associated with PMTV infection in phytohormone-related pathways. (a) Bubble plots showing significantly enriched, upregulated functional terms from GO, KEGG, and PANTHER ontology analyses. (b) Heatmaps showing expression patterns of genes with each enriched functional category, identified as responsive to PMTV infection through DGE analysis. The x-axis indicates the the time points of sample collection post-pathogen inoculation across different potato cultivars. JA: jasmonic acid, ET: ethylene, ABA: abscisic acid, AUX: auxin, BR: brassinosteroids, SH: Shepody, RB: Russet Burbank, PR: Premier Russet.

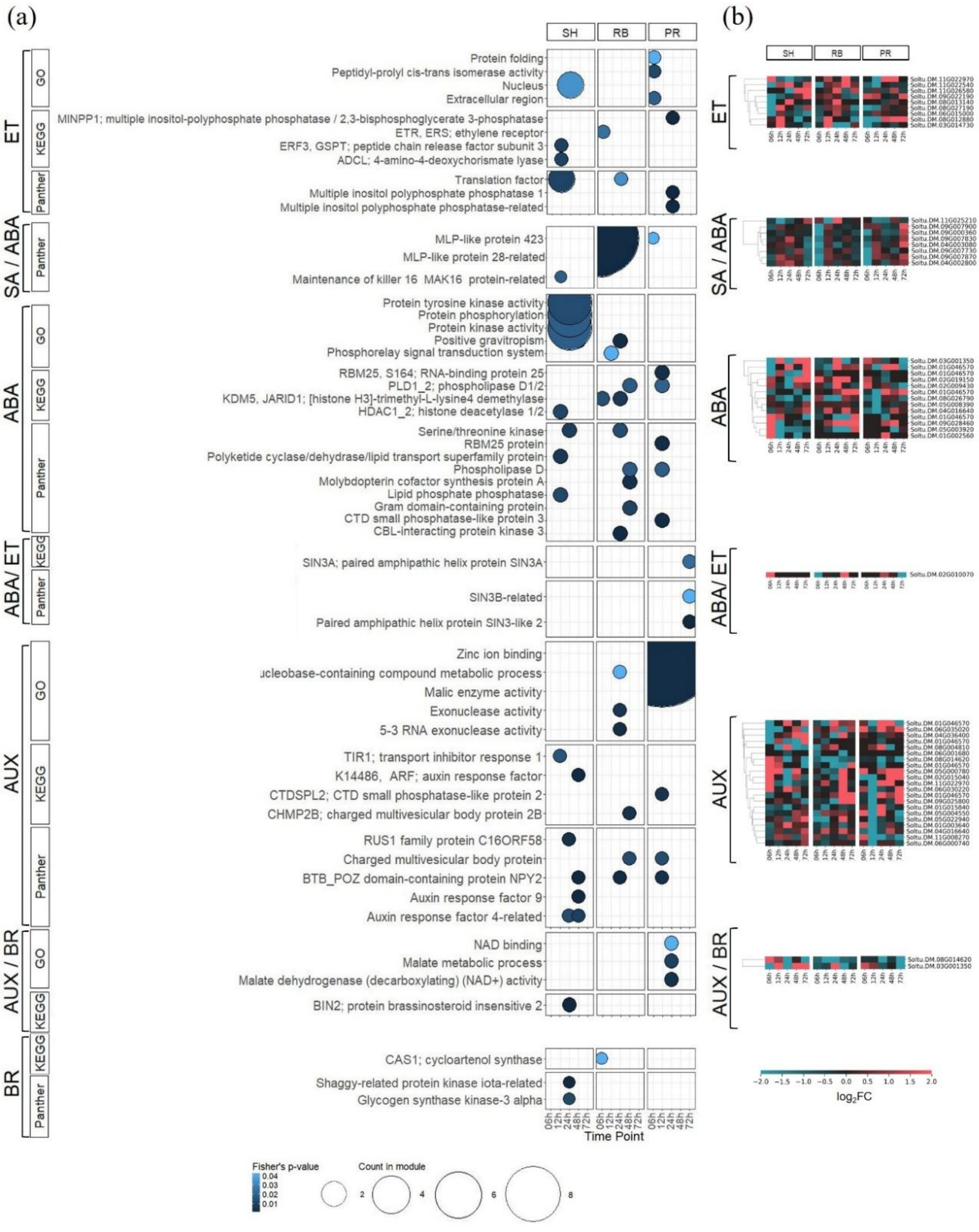

Supplementary Figure S25. Functional enrichments of downregulated DEGs associated with PMTV infection in phytohormone-related pathways. (a) Bubble plots showing significantly enriched, upregulated functional terms from GO, KEGG, and PANTHER ontology analyses. (b) Heatmaps showing expression patterns of genes with each enriched functional category, identified as responsive to PMTV infection through DGE analysis. The x-axis indicates the the time points of sample collection post-pathogen inoculation across different potato cultivars. SA: salicylic acid, JA: jasmonic acid, ET: ethylene, ABA: abscisic acid, AUX: auxin, BR: brassinosteroids, SH: Shepody, RB: Russet Burbank, PR: Premier Russet.

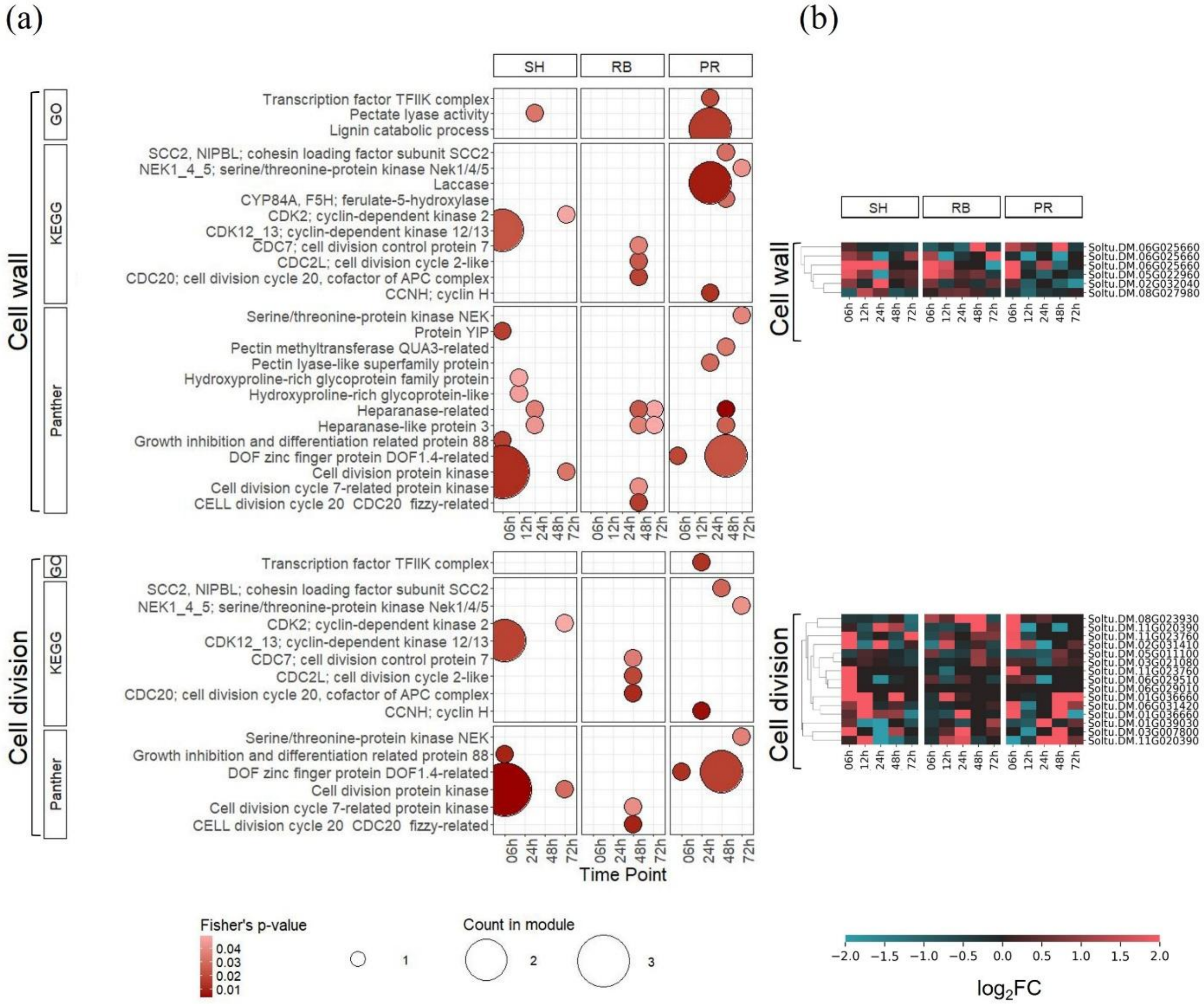

Supplementary Figure S26. Functional enrichments of upregulated DEGs associated with PMTV infection in cell wall- and cell division-related pathways. (a) Bubble plots showing significantly enriched, upregulated functional terms from GO, KEGG, and PANTHER ontology analyses. (b) Heatmaps showing expression patterns of genes with each enriched functional category, identified as responsive to PMTV infection through DGE analysis. The x-axis indicates the the time points of sample collection post-pathogen inoculation across different potato cultivars. SH: Shepody, RB: Russet Burbank, PR: Premier Russet.

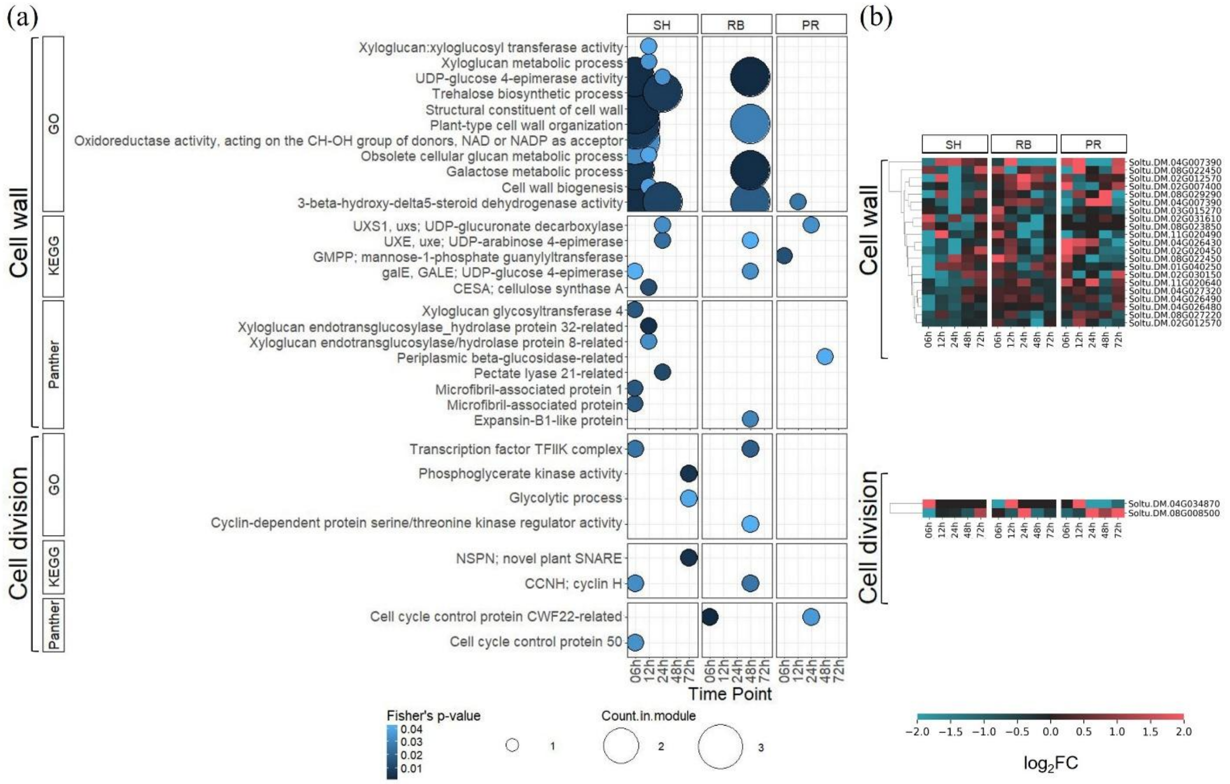

Supplementary Figure S27. Functional enrichments of downregulated DEGs associated with PMTV infection in cell wall- and cell division-related pathways. (a) Bubble plots showing significantly enriched, upregulated functional terms from GO, KEGG, and PANTHER ontology analyses. (b) Heatmaps showing expression patterns of genes with each enriched functional category, identified as responsive to PMTV infection through DGE analysis. The x-axis indicates the the time points of sample collection post-pathogen inoculation across different potato cultivars. SH: Shepody, RB: Russet Burbank, PR: Premier Russet.

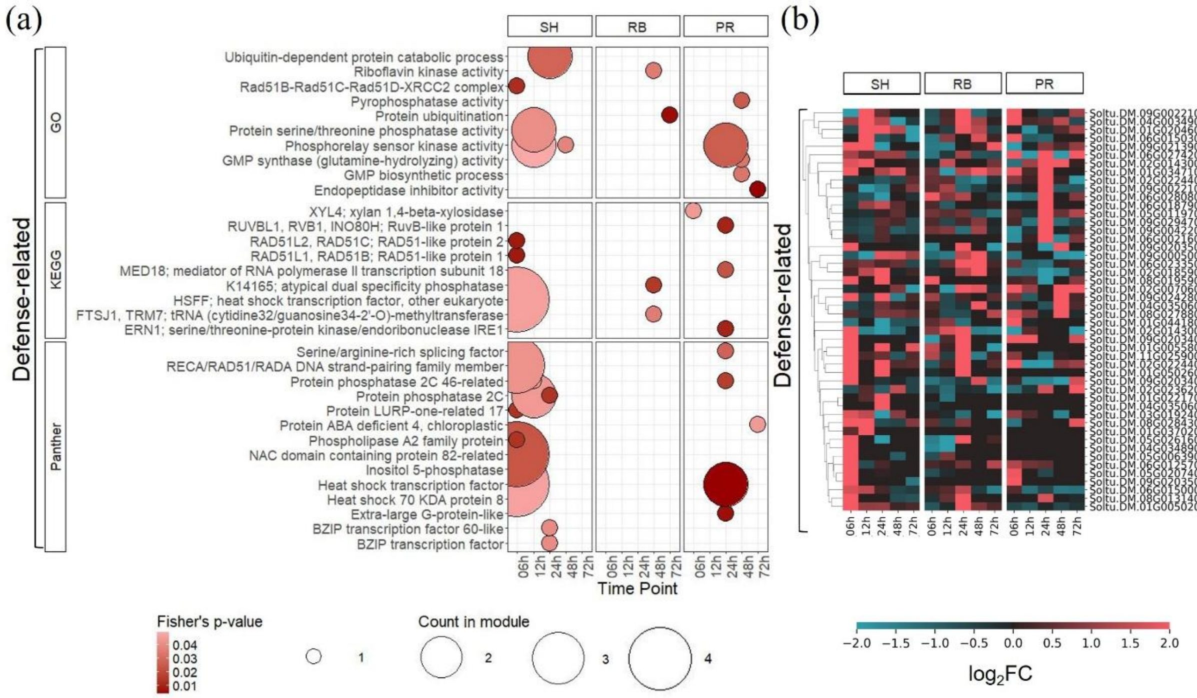

Supplementary Figure S28. Functional enrichments of upregulated DEGs associated with PMTV infection in defense-related pathways. (a) Bubble plots showing significantly enriched, upregulated functional terms from GO, KEGG, and PANTHER ontology analyses. (b) Heatmaps showing expression patterns of genes with each enriched functional category, identified as responsive to PMTV infection through DGE analysis. The x-axis indicates the the time points of sample collection post-pathogen inoculation across different potato cultivars. SH: Shepody, RB: Russet Burbank, PR: Premier Russet.

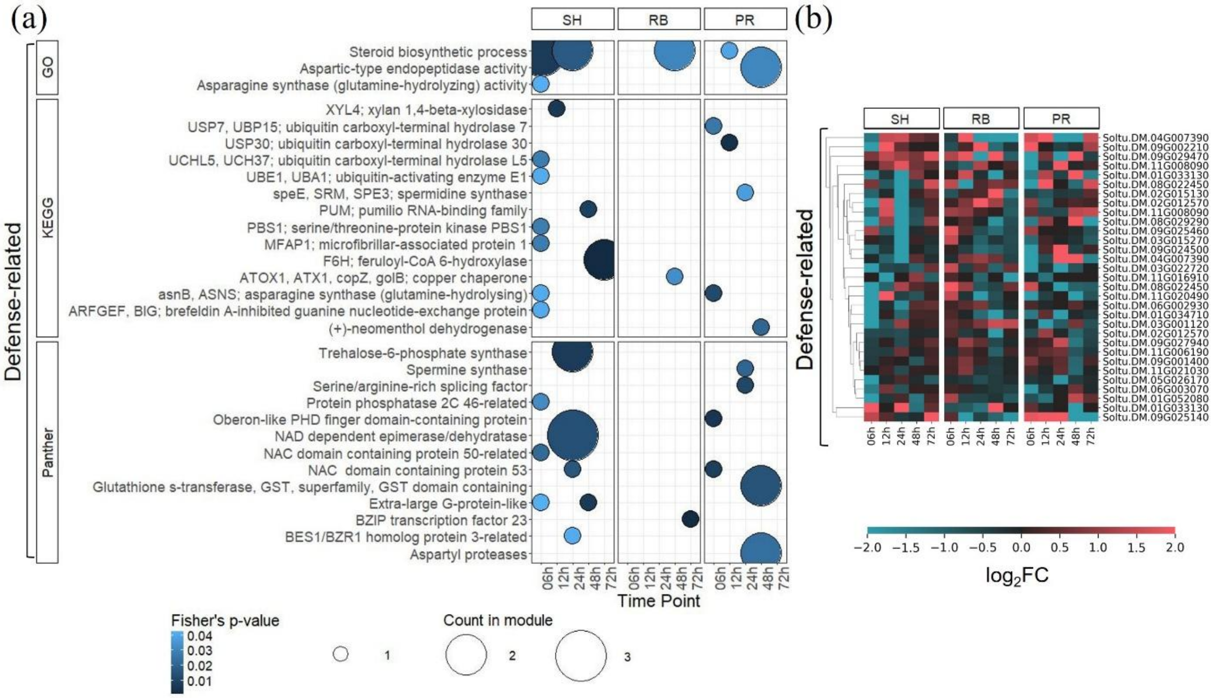

Supplementary Figure S29. Functional enrichments of downregulated DEGs associated with PMTV infection in defense-related pathways. (a) Bubble plots showing significantly enriched, upregulated functional terms from GO, KEGG, and PANTHER ontology analyses. (b) Heatmaps showing expression patterns of genes with each enriched functional category, identified as responsive to PMTV infection through DGE analysis. The x-axis indicates the the time points of sample collection post-pathogen inoculation across different potato cultivars. SH: Shepody, RB: Russet Burbank, PR: Premier Russet.

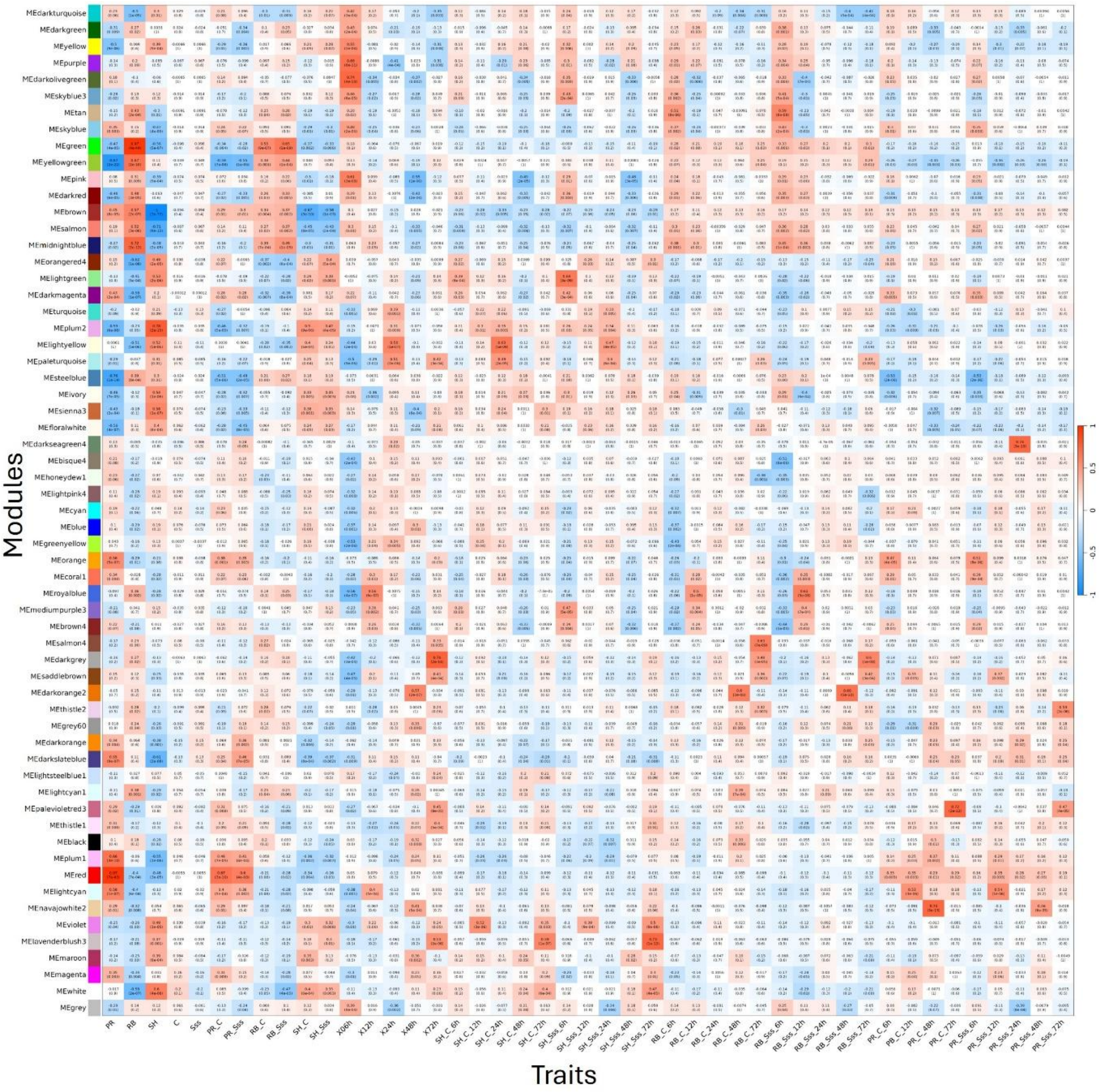

Supplementary Figure S30. Comprehensive heatmap of module–trait associations for modules identified by GCN analysis using transcriptomic data from control samples (denoted by "_C" following the cultivar acronym) and non-viruliferous *Sss*–inoculated samples (denoted by "_*Sss*"). Cultivar acronyms are SH (Shepody), RB (Russet Burbank), and PR (Premier Russet). The x-axis represents traits derived from sample metadata, and the y-axis indicates module names. Each cell displays the correlation coefficient and corresponding p-value for the association between a module and a trait. The color of the cell indicates the correlation coefficient value. *Sss*, non-viruliferous Sss inoculation; 6 h, six hours post inoculation; 12 h, twelve hours post inoculation; 24 h, twenty-four hours post inoculation; 48 h, forty-eight hours post inoculation; 72 h, seventy-two hours post inoculation.

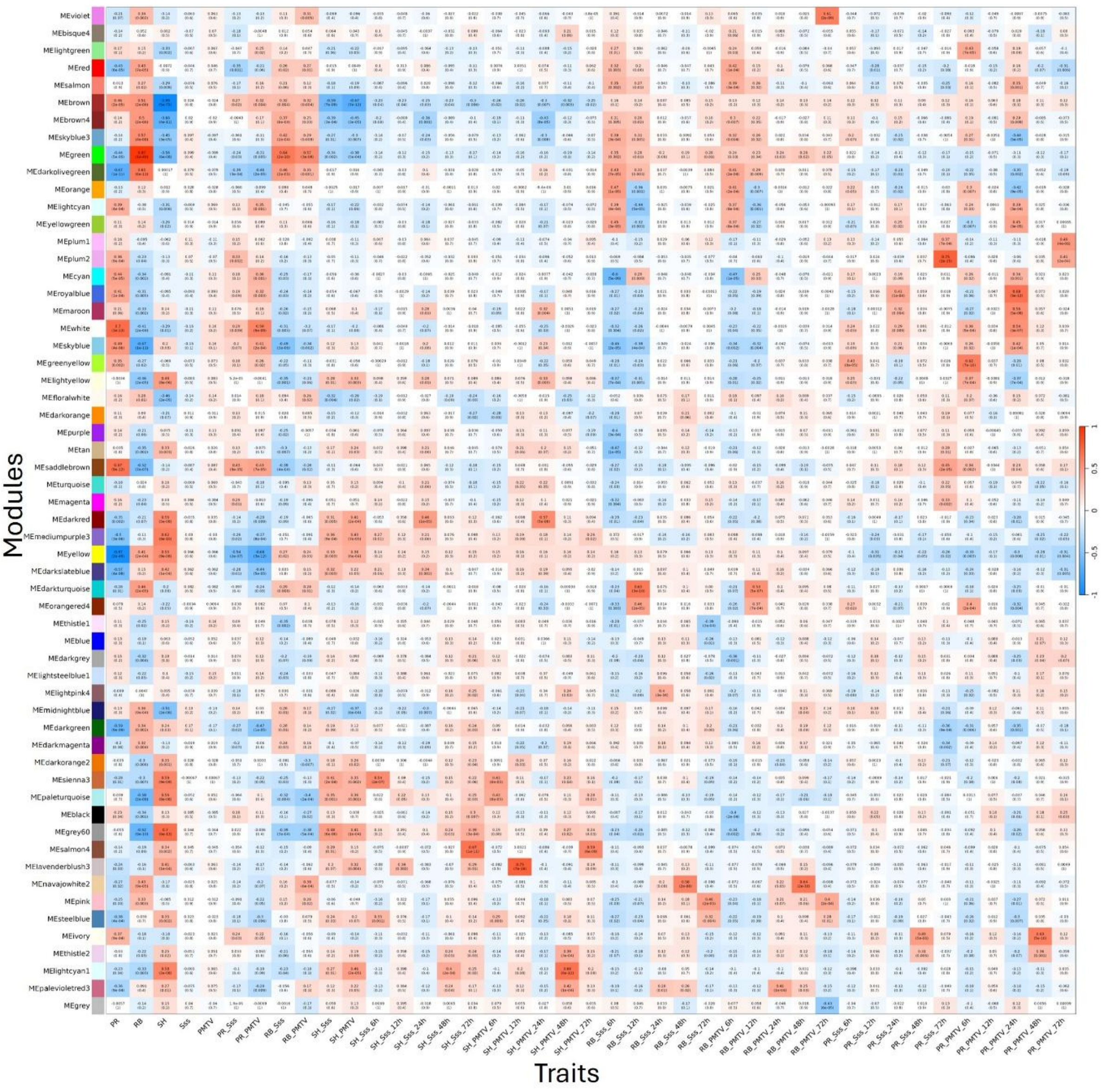

Supplementary Figure S31. Comprehensive heatmap of module–trait associations for modules identified by GCN analysis using transcriptomic data from control samples (denoted by "_C" following the cultivar acronym) and non-viruliferous PMTV-inoculated samples (denoted by "_PMTV"). Cultivar acronyms are SH (Shepody), RB (Russet Burbank), and PR (Premier Russet). The x-axis represents traits derived from sample metadata, and the y-axis indicates module names. Each cell displays the correlation coefficient and corresponding p-value for the association between a module and a trait. The color of the cell indicates the correlation coefficient value. 6 h, six hours post inoculation; 12 h, twelve hours post inoculation; 24 h, twenty-four hours post inoculation; 48 h, forty-eight hours post inoculation; 72 h, seventy-two hours post inoculation.

Supplementary Table S1: The primers used in this study. All primers listed were specifically designed for this study.

Gene ID	Primer name	Primer Sequence (5' – 3')
Soltu.DM.01G029100.3	St9100.3_Fw	CCACACACTGTTGTTATAAGAACA
	St9100.3_Rv	AATCTTCAAAGACGTTTTCGTA
Soltu.DM.01G048740.4	St8740.4_Fw	ACCTTTTCAAAGTATCATCTGGTC
	St8740.4_Rv	CCTCATCAAAGTCTATAAGCAGC
Soltu.DM.02G017990.2	St7990.2_Fw	ATCCGGCGGGATCAATTTGAT
	St7990.2_Rv	ACCAGCAGCCTCCAAGAGC
Soltu.DM.06G000540.1	St0540.1_Fw	TGAAGCCCAAAAATTTGAACCTG
	St0540.1_Rv	CGCCACGATTGCATCATCAG
Soltu.DM.01G035220.1	St5220.1_Fw	CCTTATGTCCACTGTTTTGAGGTT
	St5220.1_Rv	CGGTTCTTGCTTCTGCCACA
Soltu.DM.06G016680.4	St6680.4_Fw	ATACTATGCAAAATATGAGATG
	St6680.4_Rv	ATATGCTCCTCACATTTT
Soltu.DM.01G037540.1	St7540.1_Fw	GTTTTTTCTGCAGATTTTAATGAAA
	St7540.1_Rv	TGCATTGCAGAAATCCCAA
Soltu.DM.09G024180.3	St4180.3_Fw	CACCATGTTGTACAGAGATGTTGG
	St4180.3_Rv	GTCCCTCTAACCTTTTTTCCATTG

Supplementary Table S2. Core-module genes (Figure 8) that were also significantly identified as DEGs associated with *Sss* and PMTV infection. SH, Shepody; RB, russet Burbank; PR, Premier russet. Up, upregulated DEGs; down, downregulated DEGs.

Center of core-module	type	Gene	Gene name	Regulation in DEG
Soltu.DM.03G011810.2	Seed	Soltu.DM.03G011810.2	**LRR-RLK (RKF3-related)**	SH_24h_down
Soltu.DM.11G001840.2	Seed	Soltu.DM.11G001840.2	**TIR-containing NLR (TNL)**	SH_24h_down
	Second neighbour	Soltu.DM.01G030940.2	Integrator complex subunit 4 (INTS4)	PR_24h_up, SH_6h_up
	Second neighbour	Soltu.DM.01G033260.5	Acetyl-CoA carboxylase carboxyl transferase subunit alpha (accA)	PR_48h_up
	Second neighbour	Soltu.DM.01G034630.3	Mitochondrial carrier protein MTM1-like isoform X1	SH_6h_down
	Second neighbour	Soltu.DM.01G037540.1	WD repeat-containing protein 48 (WDR48, UAF1)	SH_24h_down
	Second neighbour	Soltu.DM.09G001760.2	BHLH domain-containing protein	PR_48h_down
	Second neighbour	Soltu.DM.09G022190.3	Set domain-containing protein	SH_6h_up
	Second neighbour	Soltu.DM.11G001840.3	**TIR-containing NLR (TNL)**	SH_24h_up
	Second neighbour	Soltu.DM.11G012500.1	Proteinase inhibitor type-2 TR8	SH_6h_up
	Second neighbour	Soltu.DM.11G026370.4	NOT transcription complex subunit VIP2 isoform x1-related	SH_6h_up
	Second neighbour	Soltu.DM.11G007360.1	Nuclear transport factor 2 (NTF2) family protein	SH_24h_up
Soltu.DM.11G002170.1	Second neighbour	Soltu.DM.11G018510.1	RNA polymerase-associated protein RTF1 homolog	SH_6h_down
	Second neighbour	Soltu.DM.11G018570.1	Gene of unknown function	PR_24h_up, SH_6h_up
Soltu.DM.06G004980.4	Seed	Soltu.DM.06G004980.4	ADP-ribosyl cyclase cyclic ADP-ribose hydrolase	PR_72h_up, SH_72h_down
